# The macroscopic limit to synchronization of cellular clocks in single cells of *Neurospora crassa*

**DOI:** 10.1038/s41598-022-10612-2

**Published:** 2022-04-25

**Authors:** Jia Hwei Cheong, Xiao Qiu, Yang Liu, Ahmad Al-Omari, James Griffith, Heinz-Bernd Schüttler, Leidong Mao, Jonathan Arnold

**Affiliations:** 1 Chemistry Department, University of Georgia, Athens, GA 30602 USA; 2 Institute of Bioinformatics, University of Georgia, Athens, GA 30602 USA; 3 Department of Biomedical Systems and Informatics Engineering, Yarmouk University, Irbid, 21163 Jordan; 4 Genetics Department, University of Georgia, Athens, GA 30602 USA; 5 College of Agricultural and Environmental Sciences, University of Georgia, Athens, GA 30602 USA; 6 Department of Physics and Astronomy, University of Georgia, Athens, GA 30602 USA; 7 School of Electrical and Computer Engineering, College of Engineering, University of Georgia, Athens, GA 30602 USA

**Keywords:** Differential equations, Nonlinear dynamics, Numerical simulations, Oscillators, Regulatory networks, Single-cell imaging, Stochastic networks, Systems biology

## Abstract

We determined the macroscopic limit for phase synchronization of cellular clocks in an artificial tissue created by a “big chamber” microfluidic device to be about 150,000 cells or less. The dimensions of the microfluidic chamber allowed us to calculate an upper limit on the radius of a hypothesized quorum sensing signal molecule of 13.05 nm using a diffusion approximation for signal travel within the device. The use of a second microwell microfluidic device allowed the refinement of the macroscopic limit to a cell density of 2166 cells per fixed area of the device for phase synchronization. The measurement of averages over single cell trajectories in the microwell device supported a deterministic quorum sensing model identified by ensemble methods for clock phase synchronization. A strong inference framework was used to test the communication mechanism in phase synchronization of quorum sensing versus cell-to-cell contact, suggesting support for quorum sensing. Further evidence came from showing phase synchronization was density-dependent.

## Significance

Describing and explaining the emergence of coherence in biological oscillators is a central unsolved problem in Collective Behavior^1^. Using microfluidics, the authors have experimentally described when the synchronization process happens as noisy single cell oscillators transition to the macroscopic limit of tissues and whole organisms. Using an artificial tissue created by microfluidics the authors observed how the clocks in single cells transitioned to a deterministic, macroscopic limit. This limit was refined by a second microwell device, which provided phase information about the oscillators through single cell tracking. Both microscopic single cell data together with macroscopic data integrated over the field of view on an artificial tissue were used to document the synchronization process. The macroscopic data identified two communication mechanisms that are possible with earlier macroscopic data from a variety of sources including RNA profiling, protein profiling, and physiological measurements on the clock through race tubes. A strong inference framework was used to test quorum sensing vs. a contact model of communication underlying phase synchronization of cellular clocks^2^. The approaches here provide a model for a method to use single cell data to explain emergent properties of tissues and whole organisms, such as circadian rhythms, and a test of two mechanisms of coherence between cellular oscillators at the macroscopic limit.

Collective behavior occurs on a variety of scales of biological organization, from the collective attack of viruses on bacterial cells^3^ and synchronization of clocks in single cells^4^ to collective behavior of flocks^5^, schools^6^, herds^7^, troops of primates^8^, and whole communities of organisms^9^. Some forms of collective behavior lead to synchronized oscillations, whether the system is cells synchronizing their clocks or fire flies synchronizing their flashing^10^. A fundamental problem in collective behavior is understanding the synchronization of biological oscillators^1^. The focus here is on the phase synchronization of clocks in single cells^4^; the problem of understanding synchronized oscillators arises in the study of other signaling systems as well^11,12^. At the single cell level there is substantial stochastic intercellular variation in the phase of cellular clocks^13^, but as these cells transition to the macroscopic limit of 10^7^ cells per milliliter (ml), the clocks become synchronized and display coherent circadian rhythms on the macroscopic scale of 10^7^ cells/ml^14,15^. Our goal here is to understand both experimentally and theoretically how this phase transition to synchronized behavior takes place in moving from cells to tissues to whole organisms^16,17^.

There are a variety of theories on how this transition to the macroscopic limit takes place. One hypothesis is that some form of cellular communication, such as quorum sensing^18–20^ or cell-to-cell contact^21^, allows the clocks in different cells to synchronize^22^. Models have been proposed for how this might happen^23–25^. A second possibility is that stochastic intracellular noise plays a positive role in synchronization^26^. In previous work it has been demonstrated that stochastic intracellular noise can lead to periodic behavior^27,28^, but these models do not address subsequent synchronization of oscillators. There is a possibility that noise could play a positive role in synchronizing the cellular clocks with respect to their phase when genetically identical cells share a common random environment as a synchronizing agent^29^. This hypothesis converges on a physical hypothesis known as Stochastic Resonance^30^, in which stochastic intracellular noise helps to solidify periodic behavior as well as oscillator synchronization. One of the earliest examples of invoking Stochastic Resonance to explain the origin of the clock is in the model clock system, *Neurospora crassa*^31^. Recently in the same clock system it has been shown from single cell data that there is one stochastic resonance predicted under a variety of Light/Dark regimens^32,33^.

**Figure 1:**
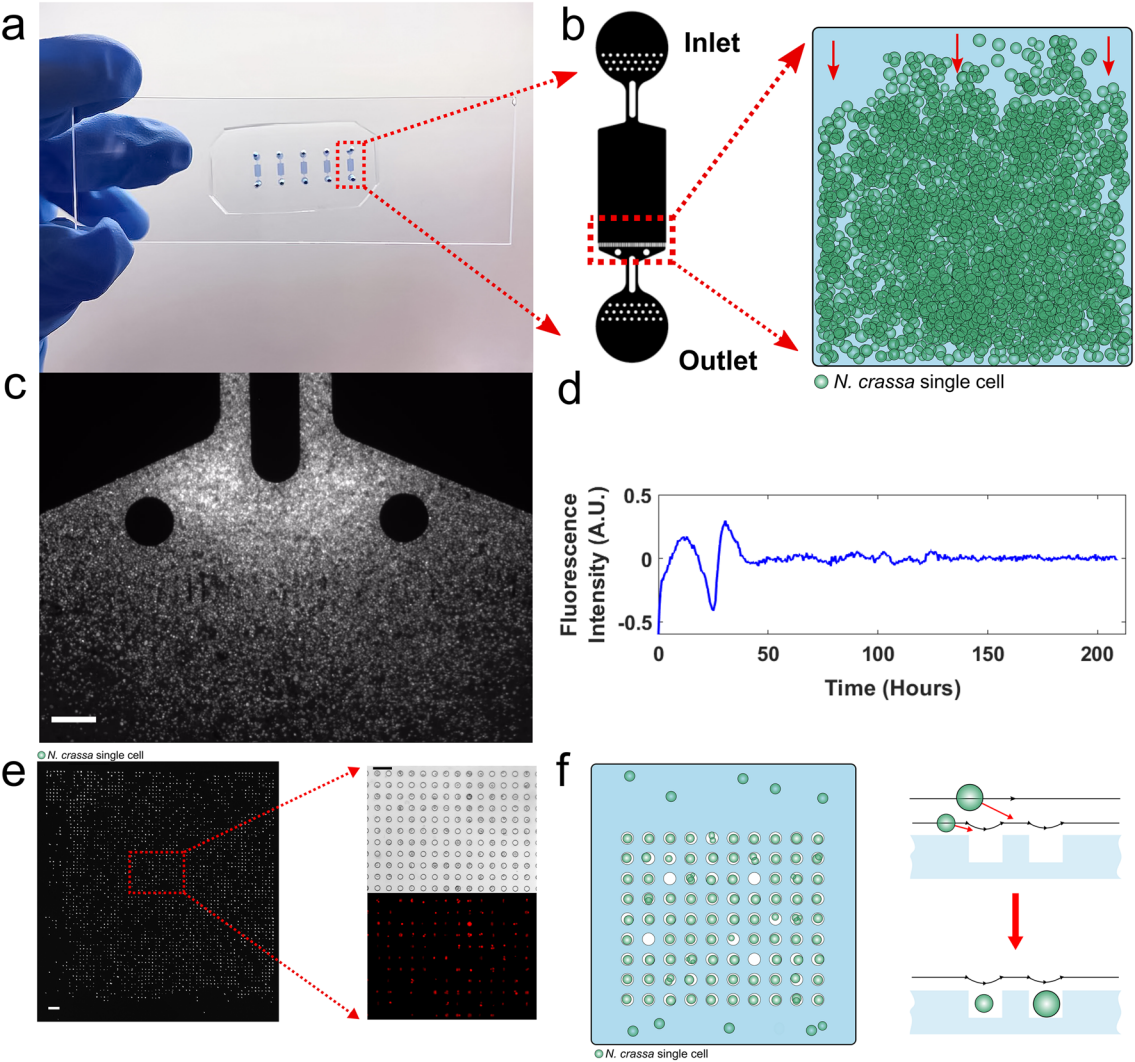
Two microfluidic devices, the “big chamber device” and microwell device, are used to characterize the synchronization of cellular oscillators on the macro-scale and micro-scale. A microfluidic “big chamber device” is developed to pack ~ 150,000 cells into an artificial tissue to examine the macroscopic limit to synchronization of cellular clocks in media 5^13^. The big chamber enables the fluorescence detection of conidial cells (strain MFNC9^42^) through an mCherry recorder driven by *clock-controlled gene-2* (*ccg-2*) promoter both in aggregate and individually^42^. Time fluorescence measurements were taken every half hour over 10 days. **(a**) An image of a microfluidic device that houses 5 big chamber devices for experiments. (**b**) Schematic of the big chamber device consisting of an inlet and outlet where cells (green circles) flow into the device from the inlet end and are gradually trapped at the barriers present at the outlet end. The dimensions of a main chamber are 1800 × 1150 × 10 (height) μm. (**c**) Fluorescence image of the cells trapped in the big chamber device. Scale bar: 50 μm. (**d**) The detrended fluorescence for around 140,000 cells is shown over 209 h. The plots were created in MATLAB_R2020B **(**https://www.mathworks.com/products/matlab.html). (**e**) A microwell microfluidic device to trap individual cells is constructed to test the quorum sensing model versus the contact model. Left: fluorescence image of MFNC9 cells in the microwell device. Scale bar: 100 μm. Right: visualization of MFNC9 cells trapped in individual microwells at 20 × magnification. (Top: bright field; bottom: fluorescence). Scale bar: 50 μm. (**f**) Schematic of cells (in green) seeded in individual microwells of 10 μm in diameter.

The advent of microfluidics^34^ allows researchers to capture and manipulate single cells to address experimentally the problem of cellular synchronization^9,35^. In order to study this interesting phase transition from cells with substantial phase variation^4^ to a state of substantial phase locking, two microfluidic platforms (Fig. 1), the “big chamber device” and microwell device, were developed. The purpose of the big chamber device was to reproduce the transition in synchronization behavior of conidial cells at the macroscopic limit of 10^7^/ml. Nakashima^36^ developed the liquid culture assay used in most molecular studies of the clock in *Neurospora crassa*^37–40^. The definition of the macroscopic limit used here throughout is reproducing the behavior of these Nakashima liquid cultures and the synchronization of the cellular oscillators in such cultures. One of the features of these liquid conidial cultures is that the clock at the macroscopic limit can only be observed over a 48 hour (h) window (Fig. 1d). On the micro-scale of single cells this limit on observation of circadian rhythms over 48 h can be removed fortunately^13^. The big chamber device also provides information about the size of the signaling molecule as discussed below. The purpose of the second microwell device (Fig. 1e–f) in contrast allows tracking of the oscillations of individual cells over 10 days and the manipulation of the conidial cell environment, such as density. In this way a more detailed study of phase synchronization can be made over 240 h.

First, the big chamber device was used to pack conidial cells of the model clock system (Fig. 1b), *N. crassa*, into one artificial tissue so that the emergence of circadian rhythms could be studied both macroscopically and microscopically simultaneously. The media was selected to minimize formation of filaments and cell fusion to simplify the modes of communication between cells^13,41^. The purpose of this report is to characterize this transition from disorder to order in an ensemble of cellular clocks in an artificial tissue. In previous work evidence was provided that single conidial cells have clocks and that most of their stochastic intracellular variation was in phase^4^. Using a second microwell device, in one experiment three hypotheses are tested: (1) phase synchronization; (2) density effect on phase synchronization; (3) contact model hypothesis. We demonstrate that phase synchronization takes no more than 829 cells or a cell density of 2166 cells per fixed area of the device and is density-dependent. Contact hypothesis is also evaluated as an alternative to quorum sensing. Two models for how this phase synchronization takes place are developed, evaluated, and compared against the aggregate behavior of cells in this artificial tissue or in a microwell device. This would allow us to make more refined predictions on when phase synchronization would occur to guide future microfluidic experiments^14^.

## Results

### Packing single cells into an artificial tissue with a “big chamber” microfluidic device

**Figure 2:**
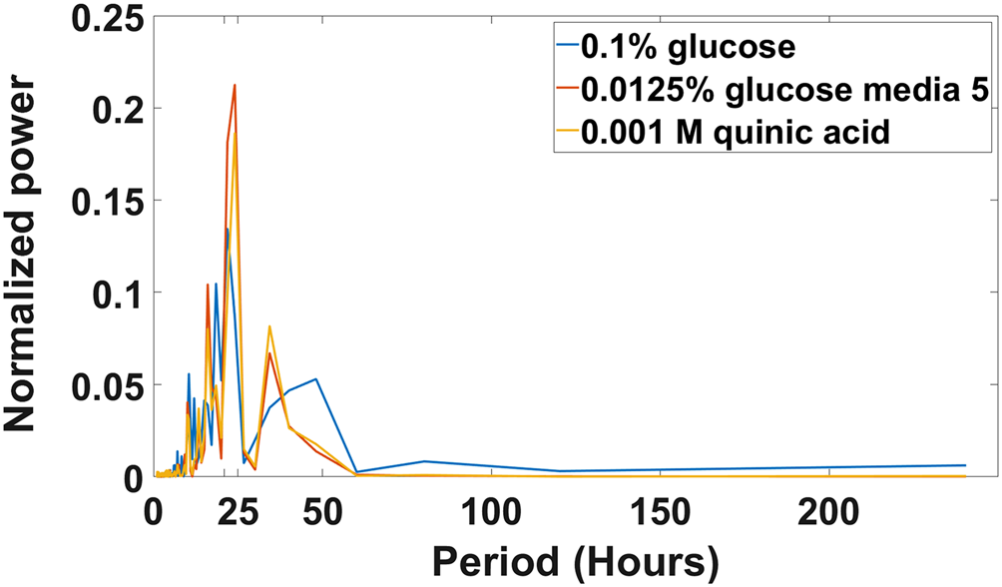
Periodogram of experiments done with MFNC9^42^ conidial cells placed in different media solutions while running a 10 day long experiment in the big chamber microfluidic device. Periodograms were generated with ~ 145,000 cells for each experiment. The plots were created in MATLAB_R2020B (https://www.mathworks.com/products/matlab.html).

In order to determine experimentally the macroscopic limit to the synchronization of cellular clocks, a “big chamber” microfluidic device with chamber dimensions 1800 × 1150 × 10 (height) μm was designed (Fig. 1a). The device trapped ~ 150,000 cells near a barrier to create an artificial tissue (Fig. 1c). Both fluorescence measurements on individual cells and aggregate measurements on five fields of view with ~ 1700 cells each were obtained. What is remarkable is that time-lapse photography (Supplementary Video) demonstrated circadian rhythms to the naked eye. The video is summarized in Fig. 1d, showing the circadian rhythm with period of 21 h in agreement to race tube experiments and liquid culture experiments beyond the macroscopic limit^14^; moreover, the oscillations are limited to 2 periods as in Nakashima liquid cultures^36^ (Fig. 1d). Aggregation of cells over fields of view and over individual cells yielded similar estimates of the period as well as the Hilbert phase curves (see “Materials and methods”) (supplementary Fig. S1). Three different media for conidial growth were tried in the big chamber with similar results (Fig. 2).

### The artificial tissue has about ~ 150,000 cells and places an upper limit of 13.5 nm on a hypothesized quorum sensing signal molecule’s radius for cellular clock synchronization

**Figure 3:**
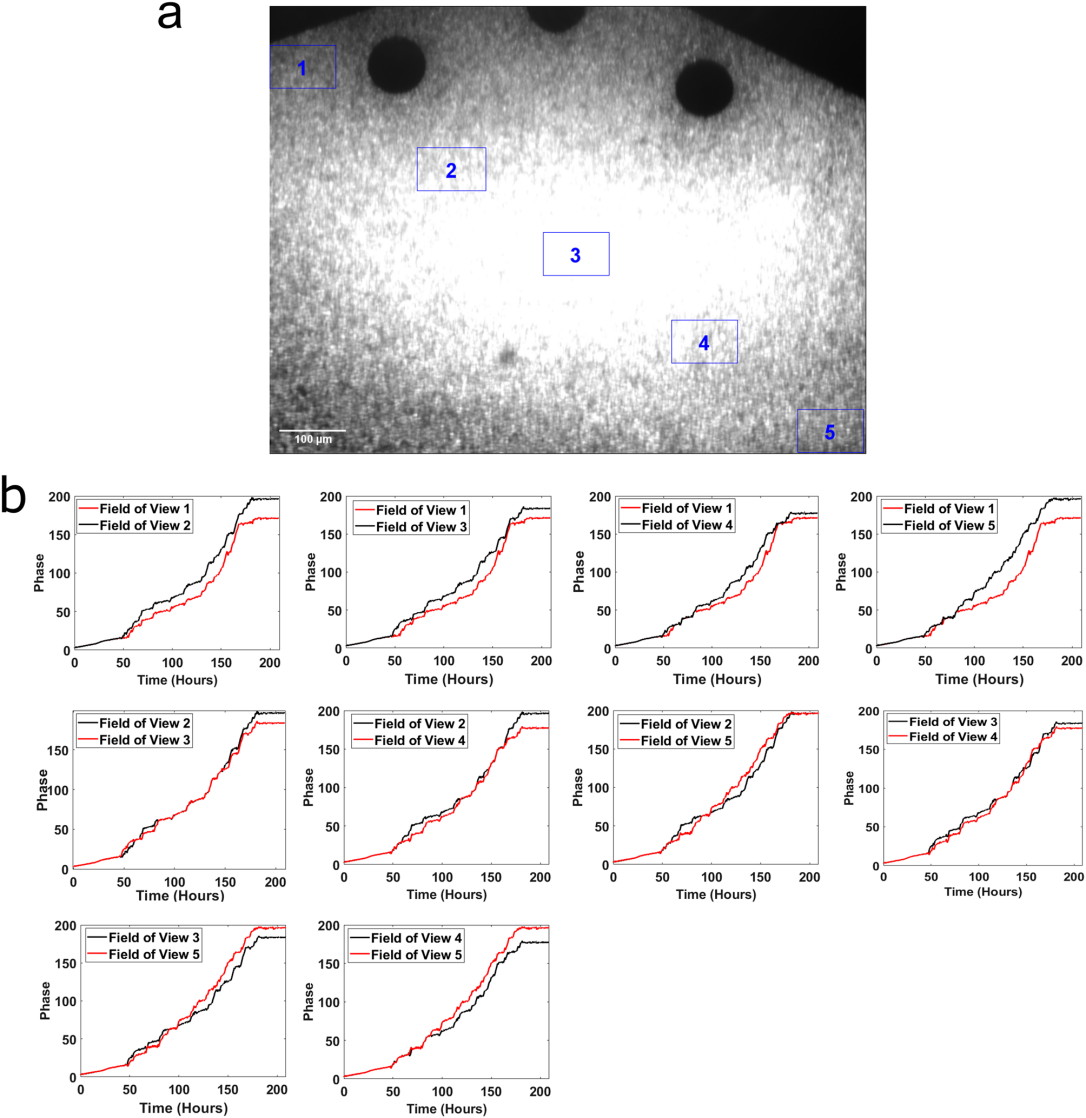
Pairwise phase trajectories of 5 fields of view in a transect across the artificial tissue in the big chamber device are highly correlated. Cells were grown in Media 5^13^. (**a**) Fields of view are shown in the artificial tissue. Each field of view contains ~ 1700 cells. (**b**) The phases between all pairs of fields of view are graphed over 10 days in the big chamber device^43^, and their computation is described in “Materials and methods”. The plots were created in MATLAB_R2020B (https://www.mathworks.com/products/matlab.html).

From the Supplementary Video and Figs. S2 and S3 it is clear that we are obtaining synchronized oscillations by cells over the dimensions of the device (1800 × 1150 × 10 μm). This synchronous behavior is captured in the phase trajectories of a fluorescent strain MFNC9 with a mCherry recorder fused to the *clock-controlled gene*-2 promoter (ccg-2P)^42^ (see “Materials and methods” for calculating phase) across different fields of view of the artificial tissue (Fig. 3)^43^.

The fields of view (as displayed in Fig. 3a for spatial location within the tissue) were highly coherent (e.g., phase synchronized) with each other as shown in Fig. 3b) and Supplementary Fig. S2. This was measured by phase measures^43^ between different fields of view on the tissue (Fig. 3b) as well as by measures of sychronization^44^. For example, the synchronization measure known as the Kuramoto order parameter (K) between different fields of view is defined as:

$$K = \left\langle {\left| {n^{ - 1} \mathop \sum \limits_{j = 1}^{n} \exp \left( {i{\text{M}}_{j} } \right) - \left\langle {n^{ - 1} \mathop \sum \limits_{j = 1}^{n} \exp \left( {i{\text{M}}_{j} } \right)} \right\rangle } \right|} \right\rangle$$

where the brackets denote an expectation over time and ${M}_{j}$ is the phase of the jth “giant cell”. The quantity n is the number of oscillators being compared (e.g., n = 2 for two fields of view) and $i= \sqrt{-1}$. If the fields of view were perfectly synchronized, the Kuramoto K would be 1.00, and if the fields of view were unsynchronized, the Kuramoto K would be 0.00. The synchronization measure (K) observed between any two fields of view was over 0.97 (Table 1) in a transect across the artificial tissue.

**Table 1:** Measures of synchronization (K) between 5 different fields of view (FOV) along a transect through the artificial tissue in Fig. 3a exceed 0.97 using the Kuramoto K^44^.

K	FOV 1	FOV2	FOV3	FOV4
FOV1	–	–	–	–
FOV2	0.9563	–	–	–
FOV3	0.9373	0.9531	–	–
FOV4	0.9318	0.9474	0.9513	–
FOV5	0.9182	0.9262	0.9267	0.9441

**Figure 4:**
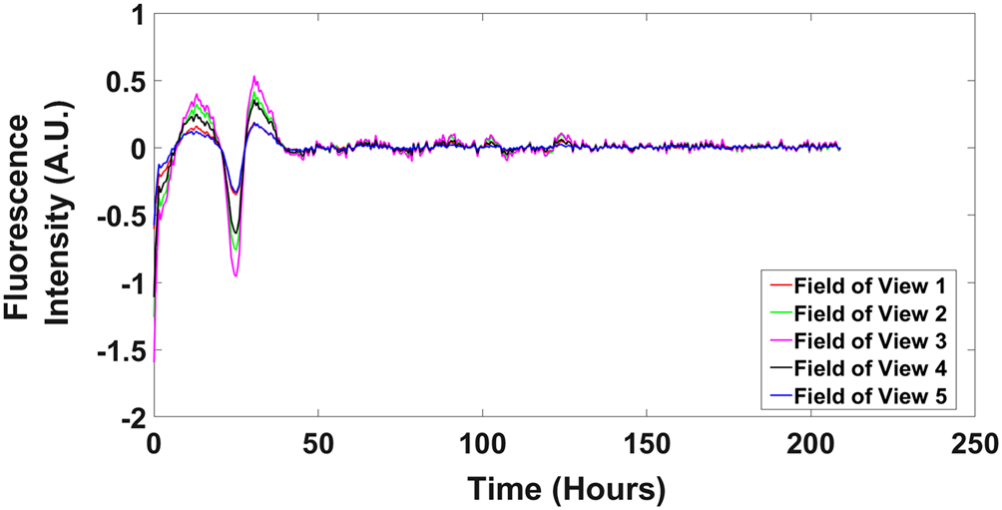
The trajectories of [CCG-2] fluorescence over all 5 fields of view in Fig. 3a aligned almost perfectly. The fluorescent intensity was normalized and detrended with a 24 h moving average over time. The plots were created in MATLAB_R2020B (https://www.mathworks.com/products/matlab.html).

The trajectories of CCG-2 recorder for different fields of view aligned with each other, showing similar fluorescent trajectories over time (Fig. 4), a result recapitulated in three other experiments done with different media conditions (Fig. S4). (The use of the term trajectory is used to invoke the connection of the data on CCG-2 with the dynamic models considered below, but the cells themselves may or may not be moving). All of these views on the phase at different locations in the tissue suggest a high degree of phase synchronization across the tissue over an 1800 × 1150 μm area (Supplement Fig. S3). A video is available showing how a quorum sensing signal in a model will synchronize cells in a tissue completely over time^4^. As the phase evolves, there is a fan shape in the spread of phase curves and averages over single cells^33^ (Supplement Fig. S3).This can be explained by stochastic intracellular variation that will result in phase variation, as well as a quorum sensing signal that synchronizes cells to the phase mean.

If this synchronization is enabled by a chemical signal diffusing in the media between cells in the artificial tissue, then diffusion theory can be used to calculate an upper limit on the size of the communication signal (See “Materials and methods”) of 13.05 nm. This includes the possibility of the signal being a protein^45^.

### A quorum sensing deterministic model predicts circadian oscillations of the artificial tissue at the macroscopic limit

**Figure 5:**
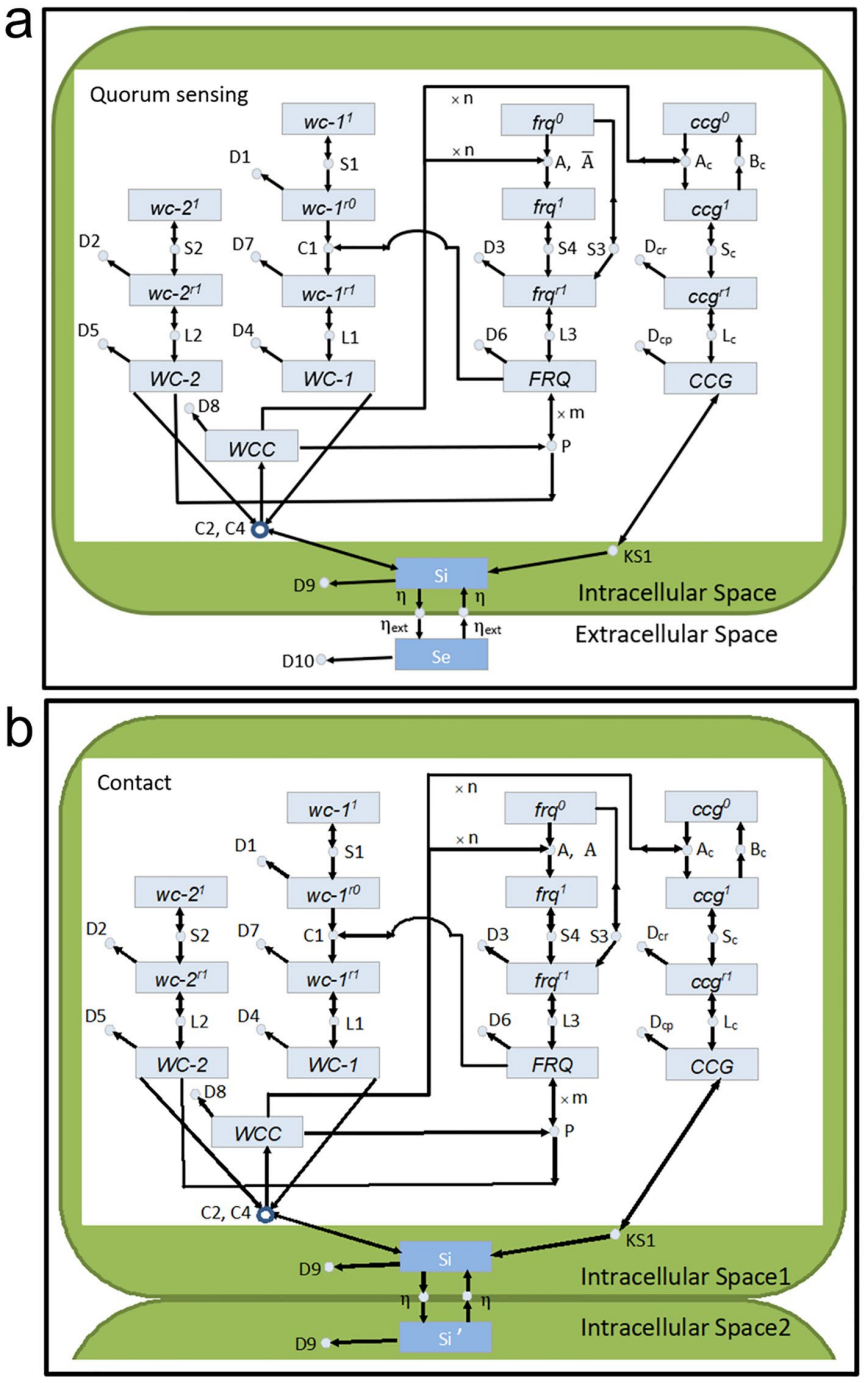
Quorum sensing and contact models for synchronizing clocks in single cells: (**a**) quorum sensing model. This is a modification of Fig. 4a in previous work^13^; (**b**) contact model.

As the macroscopic limit is approached, the full stochastic network describing the clock in single cells goes to a deterministic limit^33^, and a deterministic model can be used to describe the behavior of the clock under a quorum sensing hypothesis^13^. Each field of view that contains around 1700 cells (Fig. 3a) can be thought of as one giant cell. The molecular counts of genes and their cognate products are large in number with little stochastic intracellular variation in molecular counts of species in Fig. 5. Under the quorum sensing hypothesis, the clock reaction network is specified in Fig. 5a. This clock reaction network has a substantial body of empirical support at both the macroscopic and microscopic levels^4,13,14,32,33,46–48^. The three clock mechanism genes are *white-collar-1* (*wc-1*), *white-collar-2* (*wc-2*), and *frequency* (*frq*). The genes *wc-1* and *wc-2* are the positive elements in the clock network, and the *frq* gene is the negative element^49^. Meanwhile, the gene *frq* encodes the oscillator^37^. The concentration of the encoded protein FRQ, provides to the cell, the time of day. The FRQ protein is the pendulum on the clock, while the transcription factor complex WCC = WC-1/WC-2 is the hand that starts the pendulum FRQ moving^38^. The negative effect by FRQ occurs by its action as a cyclin to recruit a kinase/phosphatase pair to deactivate WCC^40^. This results in a negative feedback loop that explains in part how the clock mechanism produces oscillations^46^. The description of the dynamics of the clock mechanism genes and their encoded products have been identified in earlier work^46^.

In addition to the clock mechanism genes, there are two *clock-controlled genes* (*ccg*) as outputs of the clock mechanism. The hypothetical gene *ccg* encodes the quorum sensing signal CCG, and the gene *ccg-2* encodes a hydrophobin CCG-2, whose promoter is being used as the hands on the clock mechanism^42^. The gene *ccg-2* also happens to be the best characterized *clock-controlled gene*^50^. The dynamics (e.g., rate constants) of the *clock-controlled genes* are given in previous work as well^48,51^. All of the rate constants in these pieces of the model have been identified^14^, including transcription rates denoted with an S, translation rates, with an L, and decay reactions for mRNAs and proteins, with a D. The new piece in the model with unknown parameters identified here by ensemble methods^52^ (Fig. 5) is the communication between cells involving the quorum sensing signal^13^.

The quorum sensing model rests on a “mean-field” assumption where the quorum sensing signal ${S}_{e}$ diffuses instantaneously and uniformly within the big chamber microfluidic device so that all cells experience the same concentration of the signal $\left[{S}_{e}\right]$ in the device. This assumption is supported by the data (Fig. 1d). The signal in a cell ${S}_{j}$ itself is encoded and ultimately produced by the *ccg* gene at a rate ${K}_{S1}$ and decays in the media at a rate D10 and at a rate D9 in a cell. This signal diffuses in or out of a giant cell, respectively, at a rate $\eta$ or ${\eta }_{ext}$. depending on the concentration inside ([${S}_{j}$]) or outside ([${S}_{e}$]) of the cell and on volumes of the field of view and of the cell with 8 $\mu m$ diameter^53^. This diffusion assumption about the signals has been successfully used, for example, in modeling the synctitium of nuclei of the Drosophila developing blastoderm^54^. Since the field of view and cell diameter are basically the same, the areas of the field of view and of the tissue in the field of view determine the diffusion. In previous work a reasonable way for the quorum sensing signal to interact with WCC was determined, and the interaction was argued to be a negative effect on WCC production^13^. With these assumptions the diagram in Fig. 5a specifies the following system of ordinary differential equations (ODEs)^55^ to describe the clock dynamics at the macroscopic limit:

1 $$\frac{{d\left[ {wc - 1^{0} } \right]}}{dt} = 0$$

2 $$\frac{{d\left[ {wc - 1^{r0} } \right]}}{dt} = S1*\left[ {wc - 1^{0} } \right] - D1*\left[ {wc - 1^{r0} } \right] - C1*\left[ {wc - 1^{r0} } \right]*\left[ {FRQ} \right]$$

3 $$\frac{{d\left[ {wc - 1^{r1} } \right]}}{dt} = C1*\left[ {wc - 1^{r0} } \right]*\left[ {FRQ} \right] - D7*\left[ {wc - 1^{r1} } \right]$$

4 $$\begin{aligned} \frac{{d\left[ {WC - 1} \right]}}{dt} & = L1*\left[ {wc - 1^{r1} } \right] - D4*\left[ {WC - 1} \right] - \left( {C2 - C4*\left[ {Sj} \right]} \right)*\left[ {WC - 2} \right] \\ & \quad * \left[ {WC - 1} \right] \\ \end{aligned}$$

5 $$\frac{{d\left[ {wc - 2^{0} } \right]}}{dt} = 0$$

6 $$\frac{{d\left[ {wc - 2^{r} } \right]}}{dt} = S2*\left[ {wc - 2^{0} } \right] - D2*\left[ {wc - 2^{r} } \right]$$

7 $$\begin{aligned} \frac{{d\left[ {WC - 2} \right]}}{dt} & = L2*\left[ {wc - 2^{r} } \right] - D5*\left[ {WC - 2} \right] - \left( {C2 - C4*\left[ {Sj} \right]} \right)*\left[ {WC - 2} \right] \\ & \quad * \left[ {WC - 1} \right] + P*\left[ {WCC} \right]*\left[ {FRQ} \right]^{m} \\ \end{aligned}$$

8 $$\frac{{d\left[ {frq^{0} } \right]}}{dt} = - A*\left[ {frq^{0} } \right]*\left[ {WCC} \right]^{n} + \overline{A}*\left[ {frq^{1} } \right]$$

9 $$\frac{{d\left[ {frq^{1} } \right]}}{dt} = A*\left[ {frq^{0} } \right]*\left[ {WCC} \right]^{n} - \overline{A}*\left[ {frq^{1} } \right]$$

10 $$\frac{{d\left[ {frq^{r} } \right]}}{dt} = {\text{S}}3{*}\left[ {frq^{0} } \right] + {\text{S}}4{*}\left[ {frq^{1} } \right] - {\text{D}}3{*}\left[ {frq^{r} } \right]$$

11 $$\frac{{d\left[ {FRQ} \right]}}{dt} = L3*\left[ {frq^{r} } \right] - D6*\left[ {FRQ} \right]$$

12 $$\begin{aligned} \frac{{d\left[ {WCC} \right]}}{dt} & = - n{*}A{*}\left[ {frq^{0} } \right]{*}\left[ {WCC} \right]^{n} + n*Abar{*}\left[ {frq^{1} } \right] - D8*\left[ {WCC} \right] \\ & \quad + \left( {C2 - C4*\left[ {S_{j} } \right]} \right)*\left[ {WC - 2} \right]*\left[ {WC - 1} \right] - P*\left[ {WCC} \right]*\left[ {FRQ} \right]^{m} \\ \end{aligned}$$

13 $$\frac{{d\left[ {ccg^{0} } \right]}}{dt} = - Ac*\left[ {ccg^{0} } \right]*\left[ {WCC} \right]^{n} + Bc*\left[ {ccg^{1} } \right]$$

14 $$\frac{{d\left[ {ccg^{1} } \right]}}{dt} = Ac*\left[ {ccg^{0} } \right]*\left[ {WCC} \right]^{n} - Bc*\left[ {ccg^{1} } \right]$$

15 $$\frac{{d\left[ {ccg^{r} } \right]}}{dt} = Sc*\left[ {ccg^{1} } \right] - Dcr*\left[ {ccg^{r} } \right]$$

16 $$\frac{{d\left[ {CCG} \right]}}{dt} = Lc*\left[ {ccg^{r} } \right] - Dcp*\left[ {CCG} \right]$$

17 $$\frac{{d\left[ {ccg - 2^{0} } \right]}}{dt} = - Ac2*\left[ {ccg - 2^{0} } \right]*\left[ {WCC} \right]^{n} + Bc2*\left[ {ccg - 2^{1} } \right]$$

18 $$\frac{{d\left[ {ccg - 2^{1} } \right]}}{dt} = Ac2*\left[ {ccg - 2^{0} } \right]*\left[ {WCC} \right]^{n} - Bc2*\left[ {ccg - 2^{1} } \right]$$

19 $$\frac{{d\left[ {ccg - 2^{r} } \right]}}{dt} = Sc2*\left[ {ccg - 2^{1} } \right] - Dcr2*\left[ {ccg - 2^{r} } \right]$$

20 $$\frac{{d\left[ {CCG - 2} \right]}}{dt} = Lc2*\left[ {ccg - 2^{r} } \right] - Dcp2*\left[ {CCG - 2} \right]$$

21 $$\frac{{d\left[ {S_{j} } \right]}}{dt} = - D9*\left[ {S_{j} } \right] + K_{s1} *\left[ {CCG} \right] + \eta *\left( { - \left[ {S_{j} } \right] + \left. {\left[ S \right._{e} } \right]} \right)$$

22 $$\frac{{d\left[ {Se} \right]}}{dt} = - D10*\left[ {S_{e} } \right] + \eta_{ext} *\sum \left( {\left[ {S_{j} } \right] - \left[ {S_{e} } \right]} \right)$$

For simplicity the subscript for a field of view *j* on all molecular species in (1)–(20) has been suppressed. The Hill cooperativity coefficients n and m were taken as 4 in fitting models below based on previous work^46^. This deterministic model falls in the class of transcriptional repression models^56^.

The big chamber device is not sufficient to test this quorum sensing model due to the loss of phase information between cells in each field of view within the big chamber device (Fig. 1a–d). Hence, a new microfluidic device called a “microwell device” was constructed (see “Materials and methods”) (Fig. 1e–f). There are up to 15,876 wells within this device. Each well is 10 μm deep and 10 μm in diameter to trap one conidial cell of average size. Individual cells are easily tracked over 10 days, and their phase information about their individual clocks can be recovered^43^.

**Figure 6:**
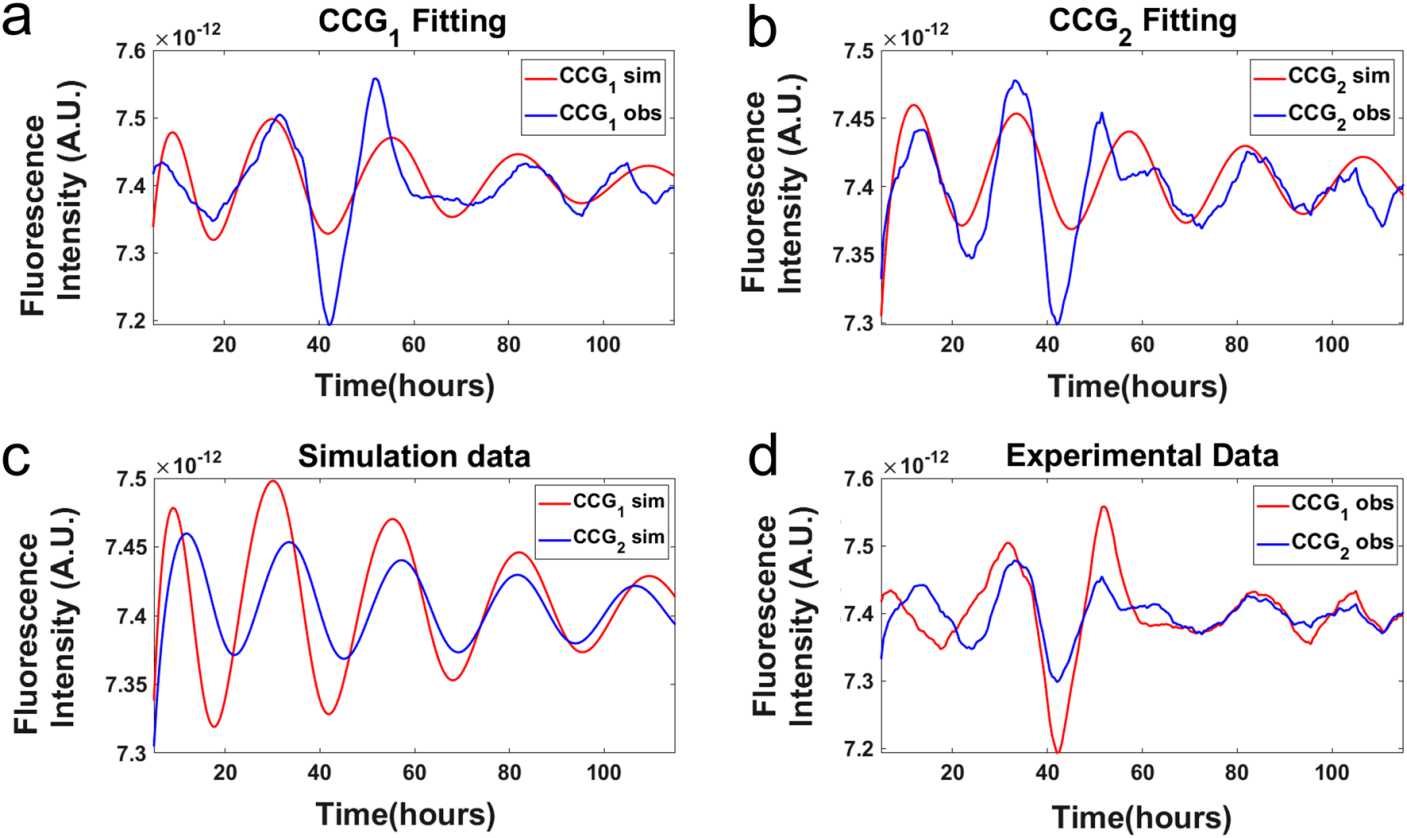
Data from CCG-2 trajectories fitted to an ensemble of deterministic model. (**a**,**b**) The trajectories of the fluorescent recorder are observed to synchronize in the first 80 h, and in the best model in the fitted model ensemble (Supplement Table S1) under quorum sensing synchronization was observed as well. Single cell trajectories were clustered into two groupings. Then the 240 single cell trajectories were averaged to create a “field of view” similar to the big chamber device. These two clusters of trajectories were then fitted by the ensemble method to the quorum sensing model in Fig. 5a^46^. (**c**,**d**) Plots of the simulation data and experimental data shows trajectories that are synchronized. The plots were created in MATLAB_R2020B (https://www.mathworks.com/products/matlab.html).

Following the example of the classic work characterizing glycolytic oscillations in *Saccharomyces cerevisiae* with a mixing experiment^57^, two populations of cells that were 12 h out of phase were then mixed together^4^, and then their synchronization was observed over time (Fig. 6d). The 240 cells were clustered by their single cell trajectories (from 0 to 30 h) into two separate clusters known as CCG_1_ and CCG_2_. An average of the cluster is taken to create an equivalent of a field of view, but the phase information of each single cell trajectory is preserved. The averages of the single cell trajectories in cluster 1 (CCG_1_) and cluster 2(CCG_2_) for the CCG-2 recorder construct^42^ are in good agreement with their model ensemble averages for the quorum sensing model (Fig. 6a–b). An examination of the observed trajectories or the expected trajectories of the CCG-2 recorder^42^ also reveals that the clusters of trajectories become synchronized in the first 80 h (Fig. 6c–d). The use of the microwell device has then refined the estimate of the macroscopic limit from ~ 150,000 cells to 15,876 cells per device area, at which phase synchronization of cellular oscillators is achieved.

As a control, these two cell populations used in the mixing experiment (Fig. 6) were loaded separately into 2 microwell devices with one population receiving an additional 12 h of light before shifting to the dark and observed over 10 days. These two isolated populations were then mixed *in computero* and clustered as in the real experiment (see “Materials and methods”). The artificial mixture was then clustered, and over 80% of the cells in the mixture on the computer were correctly assigned to their true subpopulation membership.

To test the quorum sensing hypothesis at the macroscopic limit an ensemble of deterministic models specified by Eqs. (1–22) was fitted to two CCG-2 trajectories for two clusters of cells in the microwell device (Fig. 6) (See “Materials and methods” with all tests reported below being omnibus except as noted). All parameters in the model were estimated (Table 2). The purpose of the ensemble method is to identify models consistent with the data in Fig. 6 when the number of measurements is limited, but the number of parameters (Fig. 5) is large. Ensemble methods were originally developed by Boltzmann^58,59^ and were first introduced into systems biology in 2002^52,60^. While an individual model in the fitted ensemble may be a poor predictor of the system, the average over all 40,000 models in the ensemble is quite a good predictor of system behavior (see “Materials and methods”). Not only does it allow prediction of how the system behaves (Fig. 6a,b), but it also tells us what we know and don’t know about the clock network, for example. For example, in Table 2 the estimated lifetime of the FRQ protein (1/D6 in Table 2) is about 1.7 h. The estimated value is a little shorter than the value at the macroscopic limit of 4–7 h^39^. The estimated lifetime of the stabilized *wc-1*^*r1*^ is a critical parameter in maintaining stable circadian rhythms^46^. Here its estimated lifetime is 1/D7 = 24 h (Table 2), while the measured value of 128 h was also long^46^. In general there was concordance between estimates of the rates (Fig. 5a) at the single cell level and macro scales^33^. In addition to the estimated parameters informing how the oscillations are sustained, the model identification through the standard errors (Table 2) tells us which rates are well specified by the data and which are not well specified. Both decay rates, D6 and D7, are well specified; however, there are other rates below that are not as well constrained by the data. The focus below is on the new parameters related to communication between cellular oscillators.

**Table 2:** The moments of the rate constants across the ensemble for the quorum sensing hypothesis derived from a microwell experiment.

Rate constant	Ensemble mean for each rate under quorum sensing for the microwell D/D experiment	Ensemble Standard error (SE) of rate across ensemble computed under quorum sensing for the microwell D/D experiment
A	6.946009E−03	3.313135E−06
Ā	9.969590E−02	8.297570E−05
S1	3.320978E+01	1.923567E−02
S3	1.041769E−03	2.155980E−04
S4	1.951816E+01	8.880192E−03
D1	1.164574E+00	9.405917E−04
D3	1.870605E+00	6.546756E−04
C1	1.665926E−03	1.348076E−06
L1	4.165664E+01	2.685045E−02
L3	5.276481E+00	3.137782E−03
D4	5.395039E−01	2.537757E−04
D6	5.761848E−01	2.585495E−04
D7	4.217466E−02	3.477031E−05
D8	4.741010E−05	8.957513E−06
C2	3.580439E+00	2.470929E−03
P	9.767857E+01	5.976836E−02
A_c_	1.064269E+01	5.098955E−02
B_c_	9.094971E−01	2.667601E−03
S_c_	1.490714E−03	2.502344E−06
L_c_	1.145927E−08	3.711730E−11
D_cr_	5.943974E+01	1.366934E−01
D_cp_	4.044340E−01	6.436382E−04
K_S1_	2.408651E+09	2.156671E+08
C4	1.234348E+00	5.422941E−02
η	2.038943E+00	7.304766E−01
η_ext_	2.466155E+01	2.222896E+00
D9	1.484148E+01	2.340141E−01
D10	2.374897E+00	7.021016E−01

**Figure 7:**
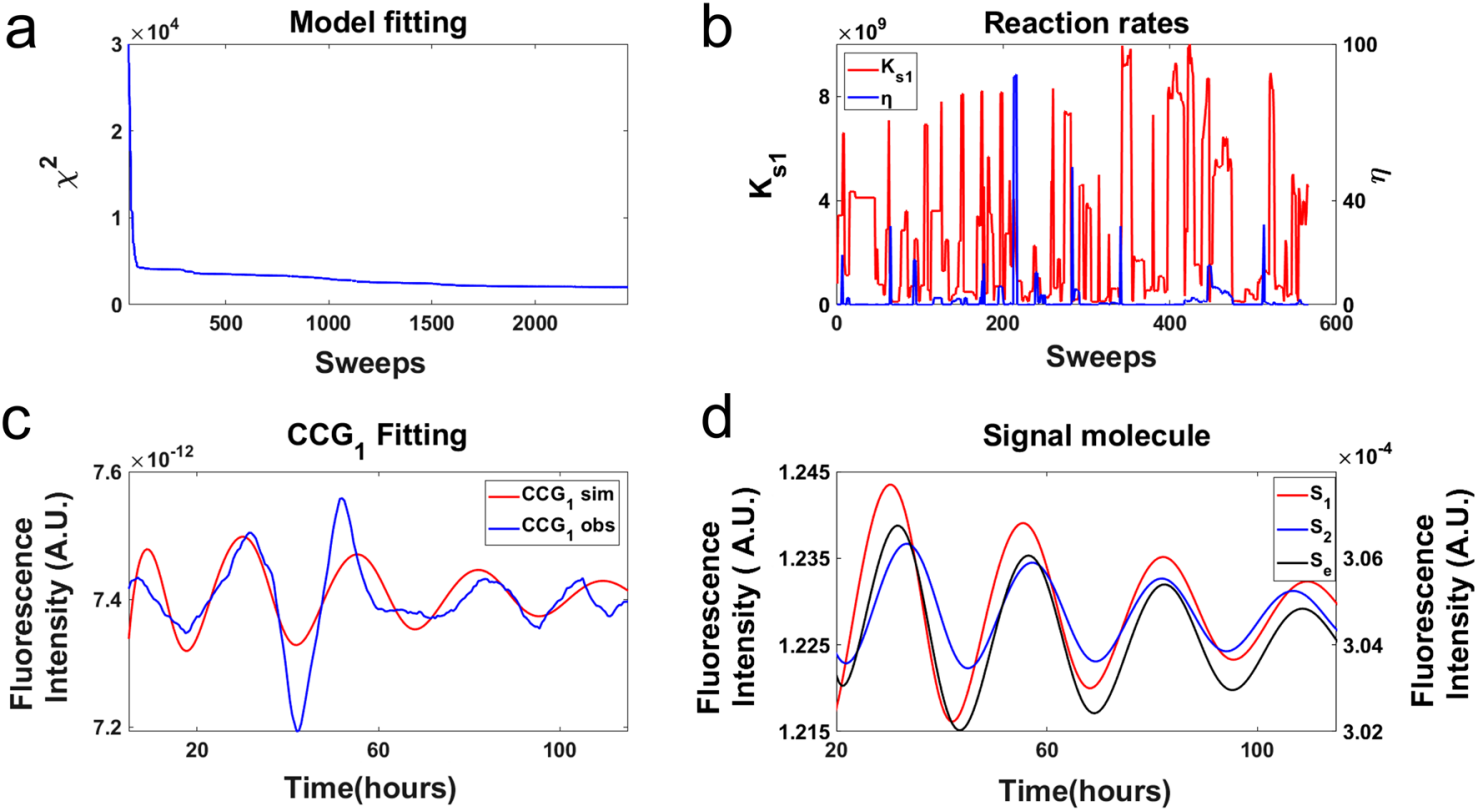
The model ensemble fitted to fluorescence of MFNC9^42^ cells with a *ccg-2* promoter in the microwell device averaged over two clusters of single cell trajectories initially with different phase (Fig. 3a) supported the quorum sensing hypothesis in a MCMC experiment. (**a**) As a control on the MCMC experiment the chi-squared statistic ${\chi }^{2}$ was plotted as a function of sweeps, i.e., a visit on average to all 71 parameters in the model. The large chi-squared statistics for sweeps 1–29 were removed to allow the rest of the chi-squared plot to be resolved. (**b**) As a second control two of the communication parameters were plotted as a function of sweeps to check that there is no trend with sweep in the MCMC experiment. (**c**) The MCMC experiment demonstrated that the measured fluorescence on one field of view fitted the quorum sensing model. (**d**) The quorum sensing signals within the two giant cells did oscillate, and they appeared to converge. The plots were created in MATLAB_R2020B (https://www.mathworks.com/products/matlab.html).

As a control on this Markov Chain Monte Carlo (MCMC) experiment, the chi-squared statistic ${\chi }^{2}$ was plotted as a function of sweeps (Fig. 7a), i.e., a visit on average of once to each of the 71 parameters (i.e., 28 rate constants and 43 initial conditions of molecular species) in the model (Fig. 5a). The MCMC experiment has equilibrated by sweep 2431 (Fig. 7a). The equilibration run yielded an ensemble of models with a good fit with ${\chi }^{2}=2016$ to n = 442 time points or $\frac{{\chi }^{2}}{n}$ = 4.56. The only departure of the model ensemble from data appears in the amplitude predicted for peak 3. So, the equilibration run was successful in converging to a well-fitting ensemble (Fig. 7a) and implies that the deterministic models collected in the accumulation run will fit the field of view data very well. As a second control, the communication parameters were plotted as a function of sweep (Fig. 7b) in the accumulation phase of MCMC. As expected, there was no systematic trend in the diffusion coefficient $\eta$ with sweep.

The MCMC experiment is summarized in Fig. 7c. The model average over the ensemble was used to predict successfully the two clusters of CCG-2 trajectories over time in each field of view, CCG_1_ and CCG_2_. The fitted ensemble predicted the cluster 1 data quite well at the macroscopic limit. Some of the behavior of the ensemble is shown for the hypothetical signaling molecule concentrations inside and outside a cell. The signal concentrations $\left[{S}_{j}\right]$ in each giant cell j (S_1_ and S_2_) of the hypothetical quorum sensing signal is clearly oscillating and driving the oscillations within each giant cell in a field of view (Fig. 7d). The media concentration of $\left[{S}_{e}\right]$ also appears to be oscillating. Our resulting model suggests that the signal concentrations in each cell appear to synchronize with the signal concentration in the media.

Summary statistics across the fitted ensemble for the 28 rate constants (their means and standard errors) are given (Table 2), and the best fitting model with initial conditions is found in Supplement Table S1. There are four key parameters in the clock mechanism^46^, the rate of activation of the oscillator gene FRQ by WCC (A), the rate of deactivation of the oscillator gene FRQ by WCC(Ā), the rate of deactivation of FRQ (P), and the rate of decay (D7) of the stabilized *wc-1* mRNA (*wc-1*^r1^). All of these values are in good agreement with their inference from previous data sets on a macroscopic and microscopic scale (Supplement Table S1).

The new information is the inference about the communication parameters, K_S1_, C4, D9, $\eta$, ${\eta }_{ext}$, and D10. The product of the rate of production of signal (K_S1_) and the effect of the signal on WCC (C4) are constant. So, only one of these two parameters can vary independently. There is limited information about the diffusion rates as seen by plotting the chi-squared surface as a function of the diffusion coefficients, $\eta$ and ${\eta }_{ext}$, with the rest of the parameters at their best values (Supplement Fig. S5). There is a lower bound on $\eta$ of around 20 and little information about ${\eta }_{ext}$. The chi-squared surface supports smaller values of ${\eta }_{ext}$ and larger values of $\eta$ for the diffusion rates. The rate of production of the quorum sensing signal (K_S1_) is large as expected^13^. The decay rate of the signal within the cell (D9) is predicted to be quite large (D9 = 10.484 h^−1^) with a lifetime of 0.1 h, and the decay rate outside of the cell (D10) is predicted to be quite large (D10 = 2.375 h^−1^) with a lifetime of 0.42 h.

**Figure 8:**
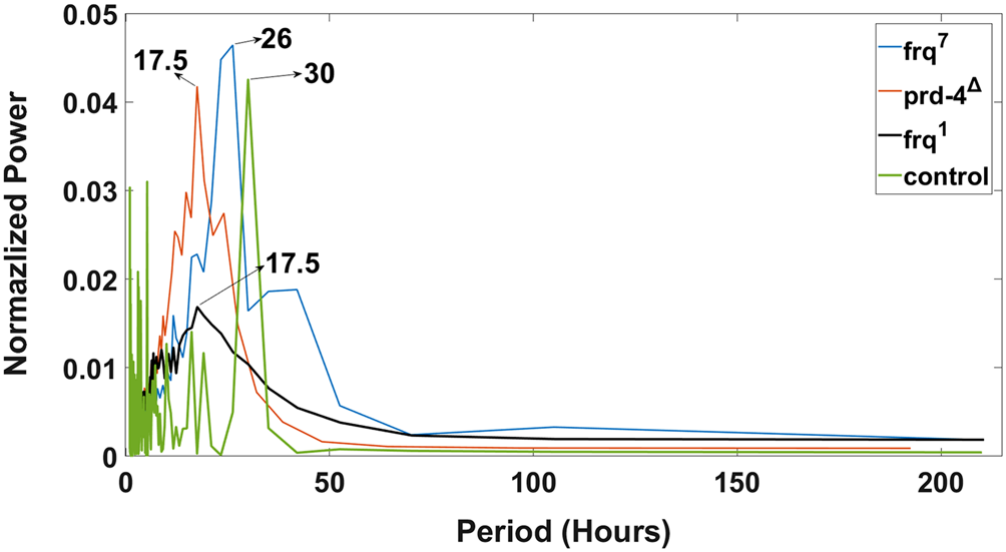
Periodogram or power spectrum of clock mutants and the noise model control. Validating the circadian signal obtained in single cells in a microwell device with clock mutants of different period. The periodograms are reported for 3 mutants in the microwell device. All detrending was done with a 24 h sliding window except for *frq*^*7*^ with its 26 h observed period. A 30 h sliding window was used for *frq*^*7*^. An artificial dataset for a sinusoid of 30 period was also created to check that the moving averaging detrending behaved appropriately to generate the simulated period of 30 h. The plots were created in MATLAB_R2020B (https://www.mathworks.com/products/matlab.html).

A variety of clock mutants exist in the biological clock of *N. crassa*. Some of the clock mutants (e.g., *period-4* (*prd-4*), 18 h; *frq-1*, 16 h)^61^ have shorter periods than MFNC9 (21 h)^13^; others have longer periods (e.g., *frq-7*, 29 h)^61^. These mutants provide an independent validation of the circadian signals seen in single cells using fluorescent strains of these mutants. Here we measured the period of these clock mutants in single cells over 10 days (Fig. 8). The resulting periods (Fig. 8) agree with mutants observed in race tubes^61^. This is another example of circadian behavior in microwell devices being consistent with measurements on the macro scale.

### A contact model is also used to predict the circadian oscillations of an artificial tissue at the macroscopic limit

An alternative to the quorum sensing hypothesis is cell-to-cell communication or a cell contact hypothesis (Fig. 5b). This mechanism operates in cell aggregation of *Myxococcus xanthus*^21,62^. Under this hypothesis only cells in physical contact (as in the tissue in Fig. 1c) can share their communication signal. Meanwhile, the cell contact model is much more straightforward because there is no signal and no decay of signal present in the medium (Fig. 5b vs. 5a). The diffusion coefficients, $\eta$ and ${\eta }_{ext}$, are replaced with one diffusion coefficient D of the signal between cells. So, there are 4 more parameters in the quorum sensing model (Fig. 5a), $\left[{S}_{e}\right]$ at time 0, D10, $\eta ,$ and ${\eta }_{ext},$ than are in the contact model and one added diffusion coefficient (D) between cells in the contact model; therefore, the contact model has 3 degrees of freedom less than the quorum sensing model. The model is captured in Fig. 5b and specifies the same system of ODEs in Eqs. (1)–(20) but with Eqs. (21–22) replaced by (23):

23 $$\frac{{d\left[ {S_{j} } \right]}}{dt} = - D9*\left[ {S_{j} } \right] + K_{s1} *\left[ {\left[ {CCG} \right] + \eta *\left( { - 2\left[ {S_{j} } \right] + \left[ {S_{j + 1} } \right] + \left[ {S_{j - 1} } \right]} \right)} \right],\quad j \ne 1,n$$

**Figure 9:**
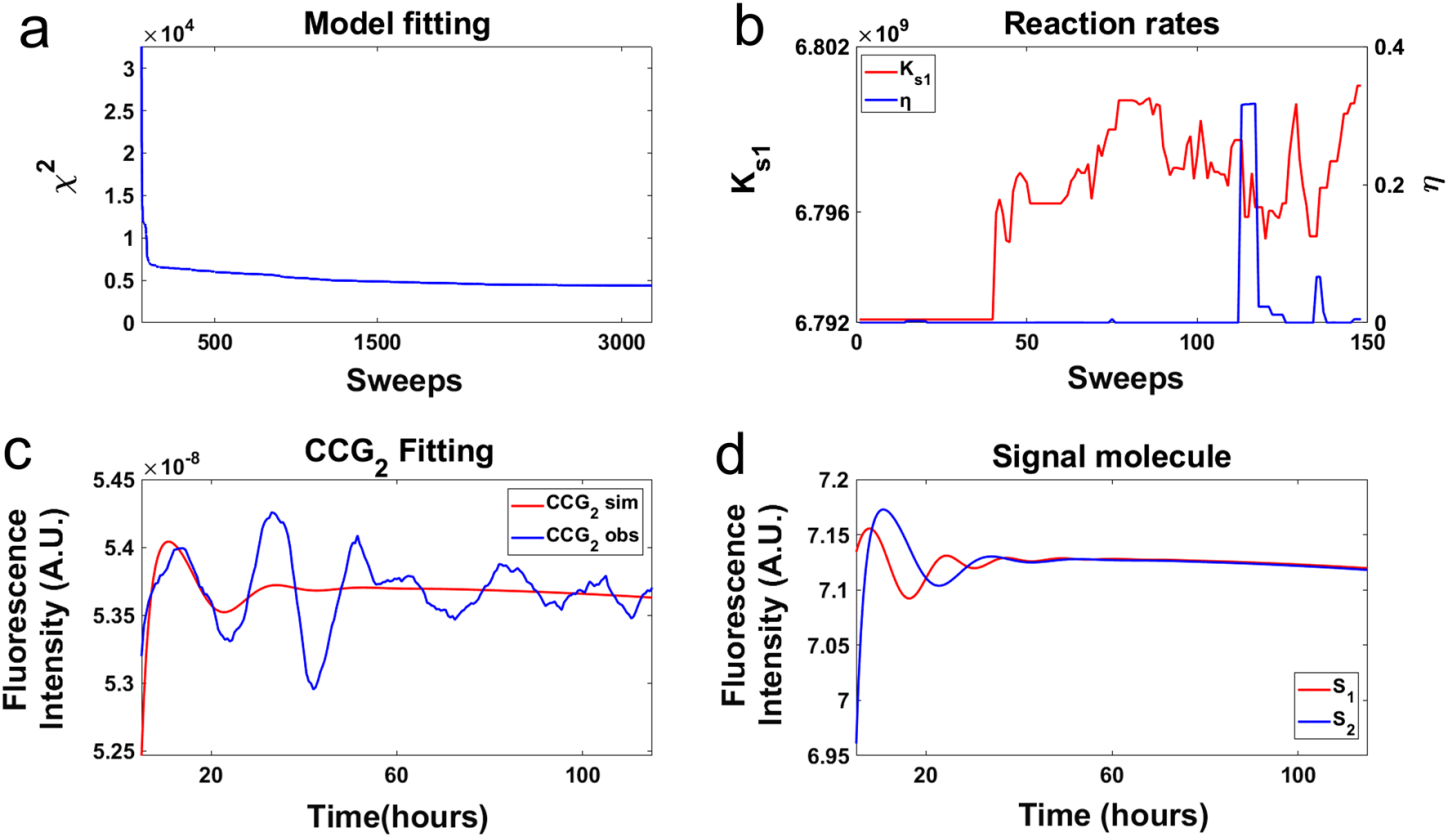
The model ensemble fitted to fluorescence of MFNC9^42^ cells with a *ccg-2* promoter in the microwell device integrated over two fields of view (Fig. 3a) did not support the contact model in a MCMC experiment. (**a**) As a control on the MCMC experiment the chi-squared statistic ${\chi }^{2}$ was plotted as a function of sweep, i.e., a visit on average to all 67 parameters in the model. The large chi-squared statistics for sweeps 1–50 were removed to allow the rest of the chi-squared plot to be resolved. (**b**) As a second control two of the communication parameters were plotted as a function of sweep to check for the presence of a trend in the MCMC experiment. (**c**) The MCMC experiment demonstrated that the measured fluorescence on one field of view fitted the contact model for one oscillation. (**d**) The quorum sensing signals (S_1_ and S_2_) within the two giant cells did oscillate in a damped way. The plots were created in MATLAB_R2020B (https://www.mathworks.com/products/matlab.html).

To test the contact hypothesis at the macroscopic limit an ensemble of deterministic models specified by Eqs. (1–20, 23) was fitted to two CCG-2 trajectories for two fields of view in the microwell device (See “Materials and methods”)^46^. As a control on this MCMC experiment the chi-squared statistic ${\chi }^{2}$ was plotted as a function of sweep (Fig. 9a). The equilibration run yielded an ensemble of models with ${\chi }^{2}=4372$ or $\frac{{\chi }^{2}}{n}$ = 9.89, after 3,187 sweeps compared to previous results^46^. So, the equilibration run was not successful in converging to a well fitting ensemble (Fig. 9a) and implies that models collected in the accumulation run do not explain the field of view data as well as the quorum sensing hypothesis. The MCMC fitting experiment is summarized in Fig. 9c. The model average over the ensemble was used to predict successfully the measured CCG_2_ trajectory over the first cycle (Fig. 9c), but not CCG_1_. Some of the behavior of the ensemble is shown for the hypothetical signaling molecule concentrations inside two giant cells. The hypothetical quorum sensing signal concentrations within a cell are clearly not oscillating in a sustained way and not driving the oscillations within each giant cell (Fig. 9d). As a consequence, the model had problems in fitting the first field of view as the communication between fields of view was not rapid enough for the convergence of the fluorescent cycles (CCG_1_ and CCG_2_). As a second control, the communication parameters were plotted as a function of sweep (Fig. 9b) in the accumulation phase of MCMC. The parameters displayed little trend, indicating further equilibration was not needed.

### Direct Test of the Contact Model versus quorum sensing hypothesis

**Figure 10:**
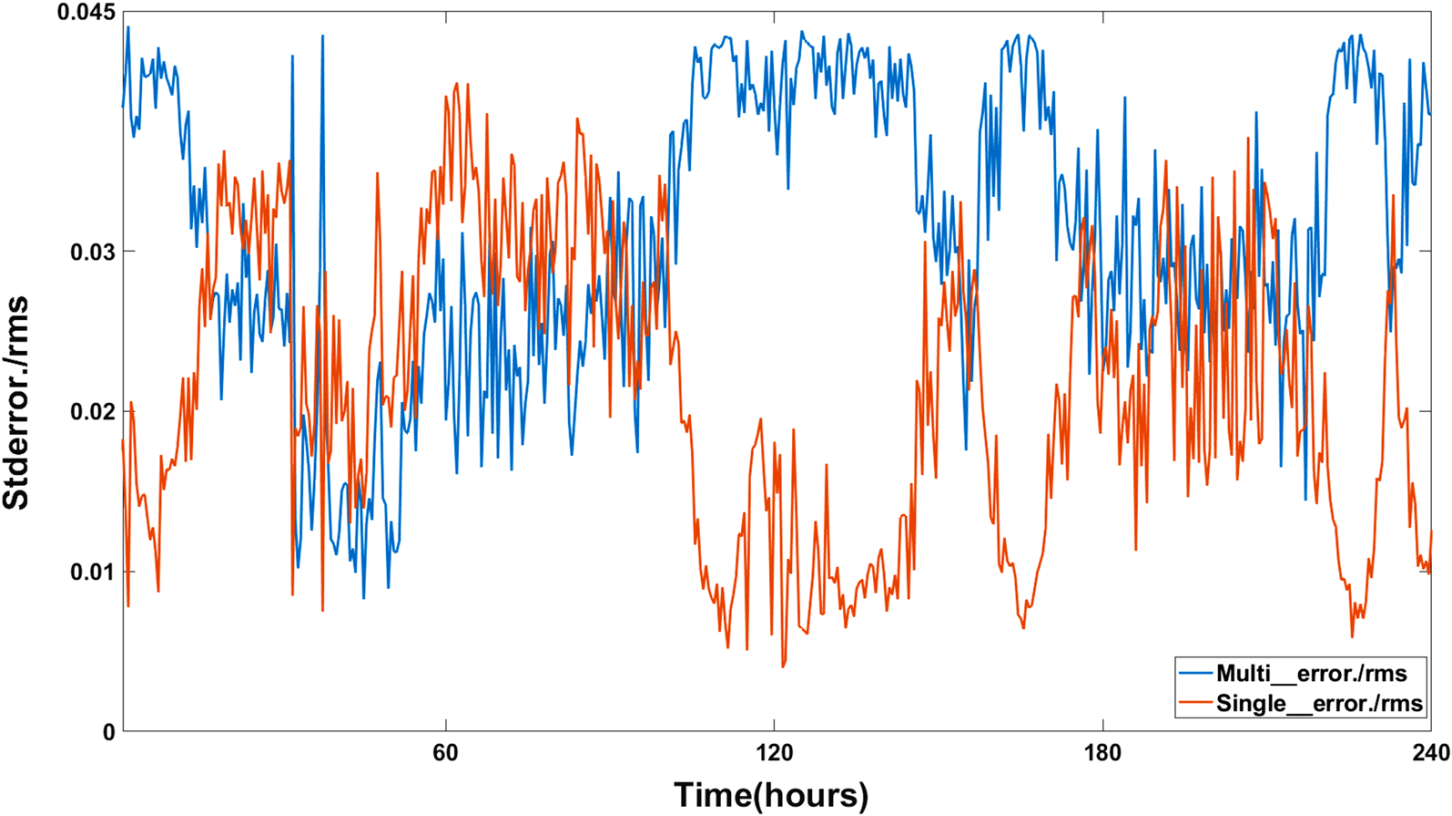
The standard error in fluorescence of single cells is significantly higher than that of multiple cells in the microwell device. There were 178 single cells in microwells, and 23 cells that were not isolated from each other. 1000 bootstrap samples were taken at each time point and used to calculate a variance (and hence standard error) at each time point. At each time point a root mean square error for single cells (X) and multiple cells (Y) was calculated with n = (178 + 23): $\sqrt {\sum \left( {\left( \frac{1}{n} \right)\left( {\sum X^{2} + \sum Y^{2} } \right)} \right)}$ and used to normalize the standard errors for single and multiple cells. Plots was created in MATLAB_R2020B (https://www.mathworks.com/products/matlab.html).

The microwell microfluidic device provides the opportunity to test the quorum sensing hypothesis against cell-to-cell communication or contact hypothesis. Based on the single cell data alone, the final chi-squared goodness of fit of the two models were significantly different (${\chi }^{2}$(contact) – ${\chi }^{2}$(quorum)) = 4373–2019 = 2354, *df* = 3, *P* < 0.0001). Relevant to distinguishing quorum sensing from a contact hypothesis, some of the microwells contain 2–3 cells, and other wells contain only 1 cell. If single cells are truly isolated and require physical contact for synchronization as in the big chamber device, the prediction is that the isolated single cells should not synchronize under the contact model. A second prediction is that under both quorum sensing and contact models there should be less variation and more synchronization in the fluorescent cells with 2 or more neighbors in a well. The results of this test are shown in Fig. 10.

The F-ratio comparing the variances across time was highly significant (F_479,479_ = 14.5144, *P* < 0.00001). The normalized standard error of single cells uniformly exceeded that of multiple cells in a well. This is consistent with there being less synchronization in single cells than between wells with multiple cells. The synchronization is also computed for the two cell populations to answer the question whether there is significant synchronization in single cells.

As a negative control the Kuramoto order parameter K was calculated on 1,644 conidial cells isolated in droplets in a flow-focusing microfluidic device^13^. The resulting Kuramoto K in 1-cell droplets in the flow-focusing device was K = 0.0322 ± 0.0007^43^. In contrast, the synchronization measure K in 1-cell microwells and multi-cell microwells were 0.7018 ± 0.0066 (n = 178 in Kuramoto K) and 0.7220 ± 0.0055 (n = 23 in Kuramoto K) respectively, which are significantly greater than the negative control. The conclusion is that single cells in microwells are showing synchronization without physical contact with other cells. This observation provides support for quorum sensing. The two Kuramoto K values for 1-cell/microwell and multiple cells/microwell are also significantly different ((z $\approx$ t_1998_ = 45.7035, *P* < 0.00001). It is possible that the slightly larger Kuramoto order parameter could be due to both a contact hypothesis and quorum sensing acting in synchrony. Hence, the contact hypothesis cannot be completely eliminated.

### Cell density and signal concentration affect cellular clock phase synchronization

With evidence for quorum sensing one prediction of the quorum sensing hypothesis was tested. A hallmark of quorum sensing is a density dependent effect on the behavior. For example, induction of Conidial Anastomosis Tubes or CATs in *N. crassa* appears to be a quorum sensing behavior, which is density dependent^64^. In *N. crassa* one hypothesis is that cell density should have an effect on communication between cellular clocks and hence their synchronization^65^.

**Figure 11:**
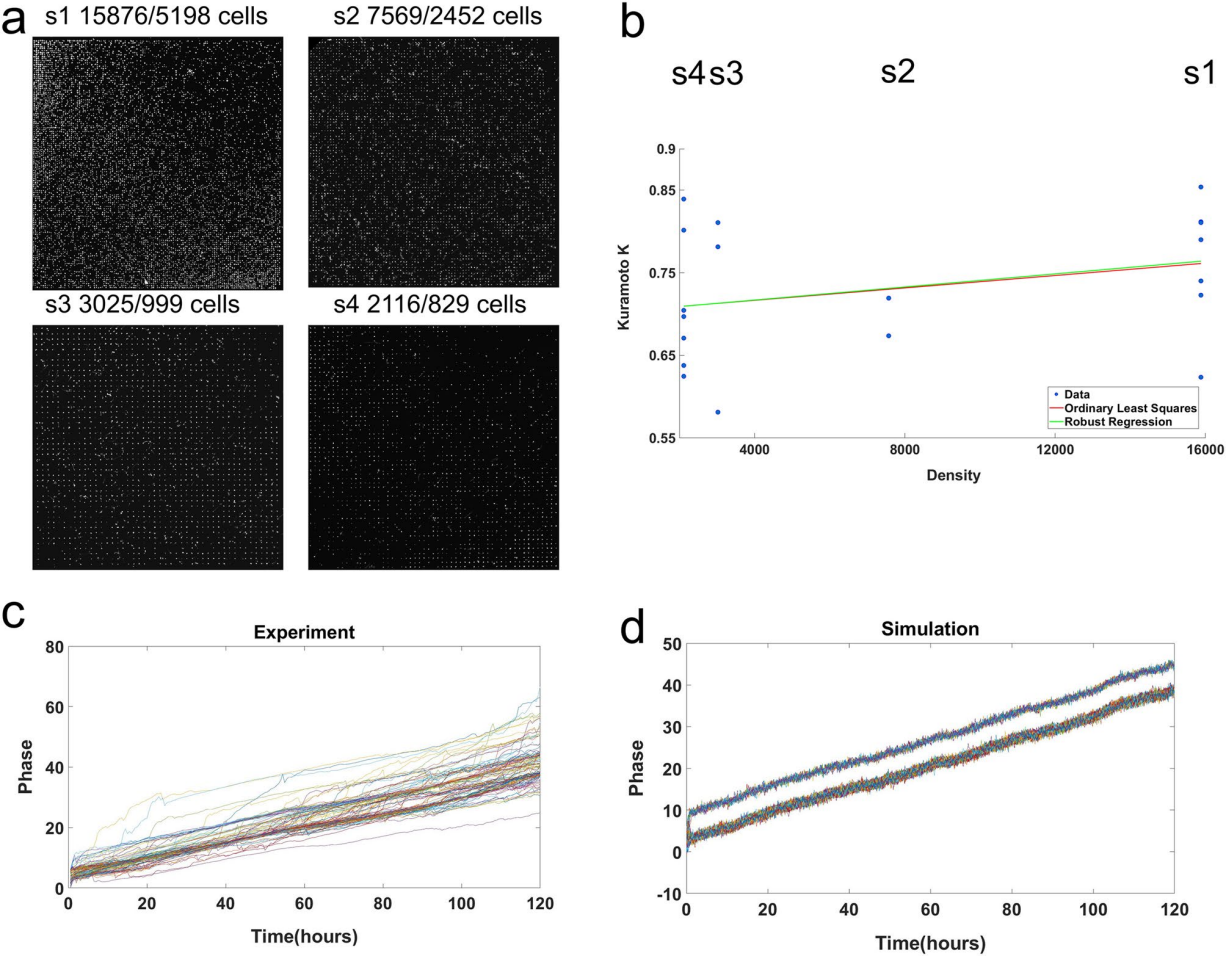
Microwell-based microfluidic chip with varying cell density gradient. (**a**) Fluorescence images of four chambers containing varying microwells of 15,876(S1), 7569(S2), 3025(S3), 2116(S4) respectively. The number of cells that was able to be tracked with Cell Profiler were 5198(S1), 2452(S2), 999(S3) and 829(S4) cells. Scale bar: 100 μm. (**b**) Robust Regression of Kuramoto K on density of cells for each microwell chamber using an M-estimator^63^ from 5 separate and independent microwell experiments. The predicted robust regression line is K = 0.70 + (3.96 ± 3.40)(10^–6^) × density (t_17_ = 1.2814, *P* = 0.1086). The test was one-sided because the expectation is that K would increase with density. Almost the same regression line was obtained with ordinary straight line regression. At least 5,000 cells were tracked in each microwell experiment. In all 5 replicates by themselves each experiment produced a positive slope between Kuramoto K and density. A sign test for a positive slope in the 5 replicates has a P = ${\left(1/2\right)}^{5}=0.03.$ Bootstrap resampling of 100 single cells was carried out to obtain the standard deviation (SE). The SE for each microwell chamber are 0.0015(S1), 0.0030(S2), 0.0039(S3), 0.0058(S4). (**c**) Plot of the experimental results of phase vs. time (5 days) with the data used in Fig. 6. (**d**) Simulation results of the ensemble method used to obtain the Hilbert phase trajectories in Fig. 6. It displays the synchronization of two different group of cells. Plots was created in MATLAB_R2020B (https://www.mathworks.com/products/matlab.html).

The microwell device in Fig. 1e–f had a cell density of 15,876 wells per area or volume of the microwell chamber, which is kept constant. The second microwell device with five chambers was constructed on the same slide with four densities of 15,876, 7569, 3025 and 2116 wells in separate chambers; the remaining chamber was reserved for mCherry beads as a control. This would allow us to measure simultaneously whether the collective behavior, such as synchronization of cellular oscillators displays quorum sensing, i.e., a cell density dependence of quorum as evidenced by cellular clock synchronization (Fig. 11). This experiment was replicated 5 times successfully to yield the relation in Fig. 11b. As the density increases, so does the synchronization of cellular clocks as measured by the Kuramoto K (Fig. 11b). In each of these 5 replicate experiments yielding the relation in Fig. 11b, the slope was always positive. By a nonparametric sign test on the 5 slopes^66^, this implies the *P*-value is ${\left(\frac{1}{2}\right)}^{5}=0.0325,$ which is significant at the 0.05 level. These measurements begin to chart out the phase transition to synchronization. The conclusion is that collective behavior of synchronization depicts quorum sensing behavior.

It is natural to ask whether or not other properties of cellular clocks have a relation to density as found in the cell density-dependent glycolytic oscillations in *S. cerevisiae*^65^. If cellular oscillators were in phase, they might be expected to reinforce the circadian signal. In fact, there also appears to be a significant relation between the average amplitude of cellular clocks (as measured by the maximum in the periodogram or power spectrum) and their density in the microwell device as they synchronize (Fig. S6a), but not with period (Fig. S6b) in contrast to glycolytic oscillations^65^.

While the quorum sensing model has a substantial body of empirical support at both the macroscopic and microscopic levels^4,13,14,32,33,46–48^, it is sometimes useful to consider a simpler heuristic model at the center of both collective behavior^10^ and statistical physics^23,67^, namely the Kuramoto model of phase synchronization, to highlight how phase synchronization is taking place. The model shares some features with our clock model of quorum sensing, such as a mean- field assumption about the quorum sensing signal. This Kuramoto model also focuses entirely on phase synchronization being described here and has been used previously to elucidate the clock model^13^.

In this model there are n oscillators with constant intrinsic frequencies ${\omega }_{i}$ and measured variable Hilbert phases ${\phi }_{i}$. Kuramoto connected these in a system of ODEs to which stochastic intracellular noise has been added:

$$\frac{{d\phi_{i} }}{dt} = \omega_{i} + K\mathop \sum \limits_{j = 1}^{n} \left( {\sin \left( {\phi_{j}- \phi_{i} } \right) } \right) +\epsilon_{i} , \quad i = 1, \ldots ,n,$$

where K is the unknown coupling constant between all of the n oscillators and ${\epsilon }_{i}$ is the stochastic intracellular white noise in the cellular clock with mean 0 and variance ${\sigma }^{2}$. A stochastic Runge–Kutta Method (SDEs)^68^ and Markov chain Monte Carlo (MCMC) were applied to identify the Kuramoto model. The ensemble method was used to fit the phase trajectories of the stochastic Kuramoto model to the measured Hilbert phase trajectories of each of the oscillators in Fig. 7 to examine the phase synchronization. In carrying out the fitting the initial Hilbert phase of each of the n oscillators at time 0 ($\phi \_i (t=0))$ and the coupling constant K were the parameters to be identified. The initial frequencies ${\omega }_{i}$ were sampled from the measured frequencies from a periodogram of isolated cells^4^. The fit was excellent with a chi-squared per data point of ${\chi }^{2}$/n = 0.69. The resulting coupling constant of K = 10.0094± 0 .0018 was substantial, which provides another line of evidence of the phase synchronization of the oscillators through a quorum sensing signal in Fig. 11d. Furthermore, the spread over time in Hilbert phases of the oscillators graphically portrays the tug of war between the quorum sensing signal to synchronize the oscillators and the noise ${\epsilon }_{i}$ decoupling them.

## Discussion

In previous work we have shown that by varying the microfluidic device and hence the cellular environment that there is the potential to test each of three hypotheses about the cause of the transition to phase synchronization of cellular oscillators^32,33^. One, there is a possibility that stochastic intracellular noise by itself can play a positive role in phase synchronization of cellular oscillators^32^. Experimental evidence for this neutral model was recently provided with a flow-focusing microfluidic device that isolated cells in droplets in previous work^32,33^. A second possibility is that a chemical signal could play a role in phase synchronization^13^ of cellular clocks^4^. Prior evidence for this hypothesis has been provided as well^4,44^. A strong inference framework was entertained for this second signaling hypothesis^2^, a signal diffusing in the media to cause synchronization^13^ versus the other alternative hypothesis involving cell-to-cell contact as a means to synchronization^21^. The final possibility is that cell cycle coupling with circadian rhythms could provide an explanation^69,70^ for phase synchronization of cellular oscillators. This hypothesis has yet to be tested in *N. crassa*. By varying the microfluidic device design each of these hypotheses can be tested^33^ and used to extract information about a putative quorum sensing signal.

A big chamber microfluidic device was designed here to create an artificial tissue that allowed observation of single cell oscillators in the macroscopic limit of 150,000 cells (Fig. 1b). This cell number was sufficient to reveal the emergence of circadian rhythms (Fig. 1d). Over the dimensions of the device a high degree of phase synchronization was observed (Fig. 3, Table 1). In fact, the dimensions of the device allowed the estimation of a bound on the putative quorum sensing signal radius of 13.05 nm. The synchronization recapitulated the behavior of Nakashima liquid cultures at the macroscopic limit^36^. It is possible that by increasing the size of the big chamber device to limit diffusion, phase variation in spatio-tempral patterns across the device could be seen^71^. In synthetic quorum sensing systems, spatio-temporal dynamics, such as waves, were observed over on a 400 μm scale, but there are other factors including the lifetime of the hypothesized quorum sensing signal S in the media (1/D10 = 0.42 h) in *N. crassa* that may have led to different behavior in the big chamber device over 1800 μm.

In order to refine the specification of the cell density at which a phase transition to synchronization takes place experimentally and to test whether collective behavior of synchronization was a quorum sensing behavior, a second microfluidic device known as a microwell device was designed to trap individual cells at varying densities. The quorum sensing model against a contact model of communication (Fig. 1e,f) was also tested. This device mimics a microtiter plate at a microscale for trapping single cells. Initially a total of up to 15,876 cells in wells in the microwell device could be individually tracked and measured for their fluorescence over 10 days (Fig. 6). Averaging over the single cell trajectories permitted the examination of phase synchronization in the macroscopic limit while preserving the phase information of individual trajectories (Figs. 7 and 9). Both the quorum sensing and contact models were fitted to experimental data. The results favored the quorum sensing model as cells were able to synchronize at a faster pace. The single cell measurements in the microwell device were also validated by the use of mutants with varying period microscopically, and the measurements in a microwell device were concordant with those at the macroscopic scale^61^ (Fig. 8). Yet, even the quorum sensing model is a simplification. Those systems displaying quorum often utilize not one signal, but multiple signals^72^. *N. crassa* quorum is likely to be more complex than hypothesized here. Some improvements in measuring phase synchronization in these new microfluidic devices should be possible with better single cell tracking methods^73^. While the microwell device is an elegant design that allows simultaneous testing of phase synchronization, density-dependence of quorum sensing, and the contact hypothesis, it has limitations. It is possible to envision other more specialized designs that more strongly test the contact hypothesis, and these designs should be pursued. Implementing the microwell design required 11 trials with 5 successes to overcome problems with number of cells tracked less than 5,000 (3 experiments failed to meet this criterion), cells growing as a failure (2), or an image stitching problem (1).

Several results support the quorum sensing hypothesis: (Fig. 7) fitting of the quorum sensing mode; (Fig. 10) greater variance in single cells vs. multiple cells in microwells; (Fig. 11) density effect on phase synchronization; (Fig. S6) density effect on amplitude. There was also one additional piece of data that was supportive of the quorum sensing hypothesis. Wells with single isolated cells in the microwell device still displayed phase synchronization (Fig. 10). This observation can be explained by the theory of the existence of a diffusible signal, but not solely with a contact model hypothesis. This result, however, does not rule out the possibility that both quorum sensing and contact could still be playing a role in chemical communication between cellular clocks.

To test directly whether synchronization of cellular clocks was a quorum sensing behavior as in CAT induction in *N. crassa*^64^, the density of cells was varied in one microwell device with multiple chambers at different cell densities (Fig. 11a). Synchronization was density-dependent as measured by the Kuramoto K order parameter and appeared to represent a second order continuous phase transition. Synchronization appeared to be occurring over the range of densities from 2166 to 15,876 wells with cellular clocks (Fig. 11a). That raises the question of how density-dependence enters into the quorum sensing model. A specific hypothesis of how this arises will be addressed with new approaches in metabolomics of living systems in real time^74^.

With several lines of evidence now for quorum sensing to explain phase synchronization between cellular clocks, the remaining question is—what is this signal molecule? From the experiment with the big chamber device (Fig. 3), an upper bound on the radius of the quorum sensing molecule of 13.05 nm was obtained. This upper bound includes the possibility that the signaling molecule is a protein as in the quorum sensing signal in the fungal pathogen, *Cryptococcus neoformans*^45^. Another possibility is that the quorum sensing signal interacts with the extracellular matrix of *N. crassa*, thereby reducing the measured rate of travel of the signal across the big chamber device and hence an increase in the estimated radius of the signal molecule. Our speculation at this time is that the second possibility is more likely and that the signal molecule is actually a metabolite. It is of considerable interest to isolate this signal molecule to explain the origin of the clock at the macroscopic scale from the behavior of single cellular clocks^4^. Isolating the quorum sensing signal will serve to test the upper bound on its estimated size of 13.05 nm.

While conidial cells are relatively easy to manipulate, a remaining challenge is the study and manipulation of the filamentous stage in the fungal syncytium with microfluidic devices^75–77^. It is very likely that by considering other life stages in the fungal synctitium other mechanisms of cellular communication will be uncovered and found to be involved in the phase transition to synchronization of cellular oscillators^78^.

## Conclusion

A “big chamber” microfluidic experiment was fabricated to demonstrate that communication existed between cells in an artificial tissue of ~ 150,000 cells. At this macroscopic limit there was a high degree of phase synchronization between cells in the artificial tissue. The dimensions of the “big chamber device” provided an upper bound on of 13.05 nm radius for the putative quorum sensing signal, which includes the possibility that the signal is a protein. In a second microfluidic experiment utilizing a microwell device housing ~ 15,876 wells, the phase of individual cells could be captured. This enabled a refinement of phase synchronization occurring with no more than 15,876 wells per chamber. A microwell with varying microwell arrays assisted in confirming that cells were able to synchronize with lower well density of 2116 per chamber in a microwell device. With the resulting single cell fluorescence trajectories of single cells in the microwell device, a strong inference framework^2^ was established to test a quorum sensing hypothesis versus a contact hypothesis for communication using ensemble methods. The ability to isolate single cells in individual wells showing phase synchronization provided strong evidence for the quorum sensing hypothesis and some information about the communication parameters that quantitate quorum sensing. Using the microwell devices, the collective behavior of synchronization was shown to be density-dependent and hence a quorum sensing behavior.

## Materials and methods

### Device design and fabrication

Microfluidic devices were made of polydimethylsiloxane (PDMS) using standard soft lithography techniques. The microfluidic “big chamber” device consisted of one inlet and one outlet for sample loading, an empty chamber with 1150 μm in width, 1800 μm in length and 10 μm in height. The microwell microfluidic device was composed of a microwell array that are 10 μm in diameter and 10 μm deep. microwell array contains an interlaced 126 × 126 grid of wells, resulting in a total of 15,876 wells. An additional microfluidic device that contained five chambers with varying microwells was fabricated and placed on one glass slide. The microwell array was 126 × 126 (S1), 87 × 87 (S2), 67 × 67, 55 × 55(S3), 46 × 46(S4) respectively.

### Strains

A *bd,ccg-2P:mCherry,A*^79^ known as MFNC9 as well as *bd,ccg-2P:mCherry,prd-4* and *bd,ccg-2P:mCherry,frq*^*7*^ , and *bd,ccg-2P:mCherry,A,frq*^*1*^ were utilized for most fluorescent measurements. A *bd,ccg-2P:mCherry,A,frq*^*7*^ and *A bd,ccg-2P:mCherry,A,frq*^*1*^ were created by the crosses MFNC9a x frq^1^,bd (FGSC 2670) and MFNC9a x frq^7^,bd (FGSC 4878), and the *prd-4* fluorescent mutant was described previously^13^.

### Microfluidic experimental setup

MFNC9 cells and related strains with the *mCherry* recorder were first placed under an LED light source (color temperature 6500 K) for 26 h in three different media: (1) media 5 described previously^13^; (2) 0.1% glucose + Vogel’s media^80^; (3) 0.001 M quinic acid + Vogel’s media. Cells were loaded into the big chamber polydimethylsiloxane (PDMS) microfluidic device (Fig. 1) using a syringe pump at a flow rate of around 5 μL min^−1^. For the microwell device, 50 μL of 70% ethanol was pipetted into the inlet, followed by priming with 50 μL of 1 × PBS supplemented with 0.1% (w/v) bovine serum albumin (BSA). This was followed by pipetting 30 μL of cell suspension. Cell concentration of 6 × 10^7^ cells/ml were used. Extra cells that were not captured in microwells were washed away with extra media. mCherry beads (Takara Bio) are loaded into one of the microwell chambers as a control for all experiments.

### Imaging and cell tracking

A CCD camera (AxioCam HRm, Carl Zeiss Microscopy, LLC, Thornwood, NY) was used to record the fluorescence intensity of cells through a microscope (Imager. M2, Carl Zeiss, Microscopy, LLC,Thornwood, NY) with a motorized x–y stage (Mechanical stage 75 × 50 R, Carl Zeiss Microscopy, LLC, Thornwood, NY) in a dark room. The microscope consists of a Colibri LED light source with continuous brightness adjustment and automatic calibration. Images were taken every 30 min with an exposure time of 900 ms over the 10 day experiment. Loss of cell viability was measured to be 20% or less over 10 days^13^. Autofocus was not used because it increased the exposure time and hence possibly photobleaching. The excitation light from a LED light source was guided through a filter set (Filter Set 60HE, Carl Zeiss Microscopy, LLC, Thornwood, NY). All experiments conducted were done in an environmental control enclosure chamber (InVivo Scientific) at a temperature of 30 °C.

CellProfiler was used to track individual cells over time^13,73^ and validated against our own MATLAB cell tracking code over time^13^ reported previously. The number of cells tracked was lower as cells that grew filaments were discarded from the tracking process. Each fluorescence time series were normalized with mCherry beads, log-detrended with a 24 h moving average^13^, and the periodogram computed^13^. The fluorescence of the field of view was obtained by integration over the field of view. In parallel for each field of view the total fluorescence was normalized with packed mCherry beads, log-detrended with a 24 h moving average, and used in deterministic model identification by ensemble methods. Prior work had demonstrated that variation in room temperature of the LED light source was 1.11% per 1 °C and highly correlated with control bead intensity^81^. Thus, normalization by the intensity of the mCherry beads removed variation in LED light source intensity”.

### Estimating an upper bound on the size of the quorum sensing molecular signal

Assuming that a quorum sensing molecule exists, we made an estimate that it would take 24 h for it to diffuse across the whole device with a size of 1800 μm. The molecular diffusion coefficient is then calculated by the following equation ${D}_{A }={L}_{a}$^2^/t_D_. ${D}_{A}$ is the diffusion coefficient of the quorum sensing molecule while *L*_*a*_ is the size of the microfluidic device where the cells are confined in, t_D_ the travel time and *L*_*a*_, the travel distance. We are able to obtain a diffusion coefficient of 2250 μm^2^/min with this following equation. Next, we used the Stokes–Einstein Equation to obtain an estimate of the upper limit of the size with $D_{A} = k_{B} T/\left( { 3\pi\upeta d_{A} } \right)$ where D_A_ is the molecular diffusion coefficient, absolute temperature T, by the Stokes–Einstein Equation, ${d}_{a}$ the diffusants molecular diameter, $\upeta$ the solvent viscosity. We were then able to obtain an upper limit of size of the quorum sensing signal as 13.05 nm.

### Calculating phase

To calculate the phase for a fluorescent series x(t), first the Hilbert transform $\tilde{x }\left(t\right)= PV \frac{1}{\pi }{\int }_{-\infty }^{\infty }\frac{x\left(\tau \right)}{t-\tau }d\tau$ was computed from the Fast Fourier Transform^82^ of x(t). The Hilbert phase ${F}^{H}\left(t\right)$ is defined as the phase angle between the Hilbert Transform $\tilde{x }\left(t\right)$ and x(t) by ${F}^{H}\left(t\right)= {tan}^{-1}\left(\frac{\tilde{x }\left(t\right)}{x\left(t\right)}\right) \mathrm{t}$o avoid discontinuities in the phase angle at $\pi$ and $-\pi$, the Hilbert phase was continuized to ${F}^{C}\left(t\right)$. The continuization was done recursively through the relation: ${F}^{C}\left(t+1\right)={F}^{C}\left(t\right)+{m}^{C}\left(t\right)2\pi$, where at each step the argument m was chosen to minimize: $D{f}_{m}=\left|{F}^{H}\left(t+1\right)-{F}^{C}\left(t\right)+2\pi m\right|$. With the continuized Hilbert Phase ${F}^{C}\left(t\right)$, the phase is defined by:$M^{C} = \frac{{\left\lfloor {F^{C} \left( {t_{1} } \right) - F^{C} \left( {t_{0} } \right)} \right\rfloor }}{{2\pi }}$ in units of cycles. An accessible description of these phase measures and code to calculate them in MATLAB are available^43^ with associated MATLAB in GitHub.

### Ensemble methods

The quorum sensing and cell-to-cell contact models specifying the ODEs in (1)–(23) were identified using a Metropolis–Hastings updating scheme^46^. Proposed solutions during the Markov Chain Monte Carlo (MCMC) were with an Adaptive Runge–Kutta solver. The equilibration stage involved 40,000 sweeps. The accumulation phase involved 40,000 sweeps.

The data sets generated during the current study are available from the corresponding authors on reasonable request.

## Supplementary Information

Supplementary Video S1.

Supplementary Information 1.
