# Supplementary material for: The macroscopic limit to synchronization of cellular clocks in single cells of Neurospora crassa
Source: Sci Rep. 2022 Apr 25;12:6750. doi: 10.1038/s41598-022-10612-2 (PMC9039089; doi:10.1038/s41598-022-10612-2)
Supplement: Supplementary file 2 — Supplementary Information 1. [file 41598_2022_10612_MOESM2_ESM.docx]

Supplementary Materials for

The macroscopic limit to synchronization of cellular clocks in single cells of *Neurospora crassa*

**Authors:** Jia Hwei Cheong^1,8^, Xiao Qiu^2,8^, Yang Liu^1^, Ahmad Al-Omari^3^, James Griffith^4,5^, Heinz-Bernd Schüttler^6^, Leidong Mao^7*^, Jonathan Arnold^4*^

**^8^**Both authors have contributed equally to this work.

**Affiliations:**

^1^Chemistry Department, University of Georgia, Athens, GA 30602.

^2^Institute of Bioinformatics, University of Georgia, Athens, GA 30602.

^3^Department of biomedical Systems and Informatics Engineering, Yarmouk University, Irbid, Jordan 21163

^4^ Genetics Department, University of Georgia, Athens, GA 30602

^5^College of Agricultural and Environmental Sciences, University of Georgia, Athens, GA 30602.

^6^Department of Physics and Astronomy, University of Georgia, Athens, GA 30602.

^7^ School of Electrical and Computer Engineering, College of Engineering, University of Georgia, Athens, GA 30602.

***Corresponding authors**: Jonathan Arnold, [arnold@uga.edu](mailto:arnold@uga.edu); Leidong Mao, [mao@uga.edu](mailto:mao@uga.edu)

Correspondence to [arnold@uga.edu](mailto:arnold@uga.edu) and mao@uga.edu

**This pdf includes:**

Table S1

Fig. S1, S2, S3, S4, S5, S6, and caption for video S1.

**Other supplementary materials include:**

Video S1

**Table S1** The rate constants for the quorum sensing (column 3) are similar to isolated single cells^1^ (column 2) and those at the macroscopic limit of 10^7^cells per ml^2^ (column 4)

| **Parameter** | **Initial Parameter values from published ensemble (column 4) computed by Parallel tempering for D/D experiment^1^** | **Best parameter values from model ensemble computed by microwell D/D experiment under quorum sensing model** | **Initial Parameter values from published ensemble (column 3) computed by Parallel tempering for D/D experiment^2^** |
| --- | --- | --- | --- |
| Number of communicating cells | 1 | 240 | ~10^7^ cells/ml |
| A | 2.56E−10 | 6.852861E-03 | 0.0313 |
| Ā | 1.589532708 | 1.013056E-01 | 0.1108 |
| S1 | 80.12566921 | 3.352361E+01 | 0.000420 |
| S3 | 0.400641074 | 1.708023E-03 | 5.47E-5 |
| S4 | 8,316.020583 | 1.932607E+01 | 1.252 |
| D1 | 1.294999006 | 1.195490E+00 | 6.607 |
| D3 | 4.382612039 | 1.855057E+00 | 0.798 |
| C1 | 0.000932789 | 1.685434E-03 | 1.047 |
| L1 | 4.777735371 | 4.268960E+01 | 94.39 |
| L3 | 0.665600817 | 5.283324E+00 | 63.93 |
| D4 | 0.08474029 | 5.428893E-01 | 0.00451 |
| D6 | 0.193685712 | 5.775316E-01 | 0.205 |
| D7 | 2.130911791 | 4.301660E-02 | 0.135 |
| D8 | 0.007744621 | 6.586861E-05 | 0.0122 |
| C2 | 1.515554675 | 3.583455E+00 | 3.322 |
| P | 2.72E−09 | 9.845228E+01 | 0.2233 |
| A_c_ | 1.86E−08 | 1.109814E+01 | 0.1293 |
| B_c_ | 2.581096866 | 9.345902E-01 | 0.6091 |
| S_c_ | 61.51499414 | 1.458434E-03 | 2.572 |
| L_c_ | 1.61524392 | 1.256038E-08 | 3.664 |
| D_cr_ | 0.150810052 | 6.049845E+01 | 0.579 |
| D_cp_ | 0.54063952 | 3.940957E-01 | 0.5536 |
| K_S1_ | -- | 4.612788E+09 | -- |
| C4 | -- | 2.646258E+00 | -- |
| η | -- | 1.445784E-05 | -- |
| η_ext_ | -- | 4.094524E-01 | -- |
| D9 | -- | 2.150455E+01 | -- |
| D10 | -- | 3.057320E-08 | -- |


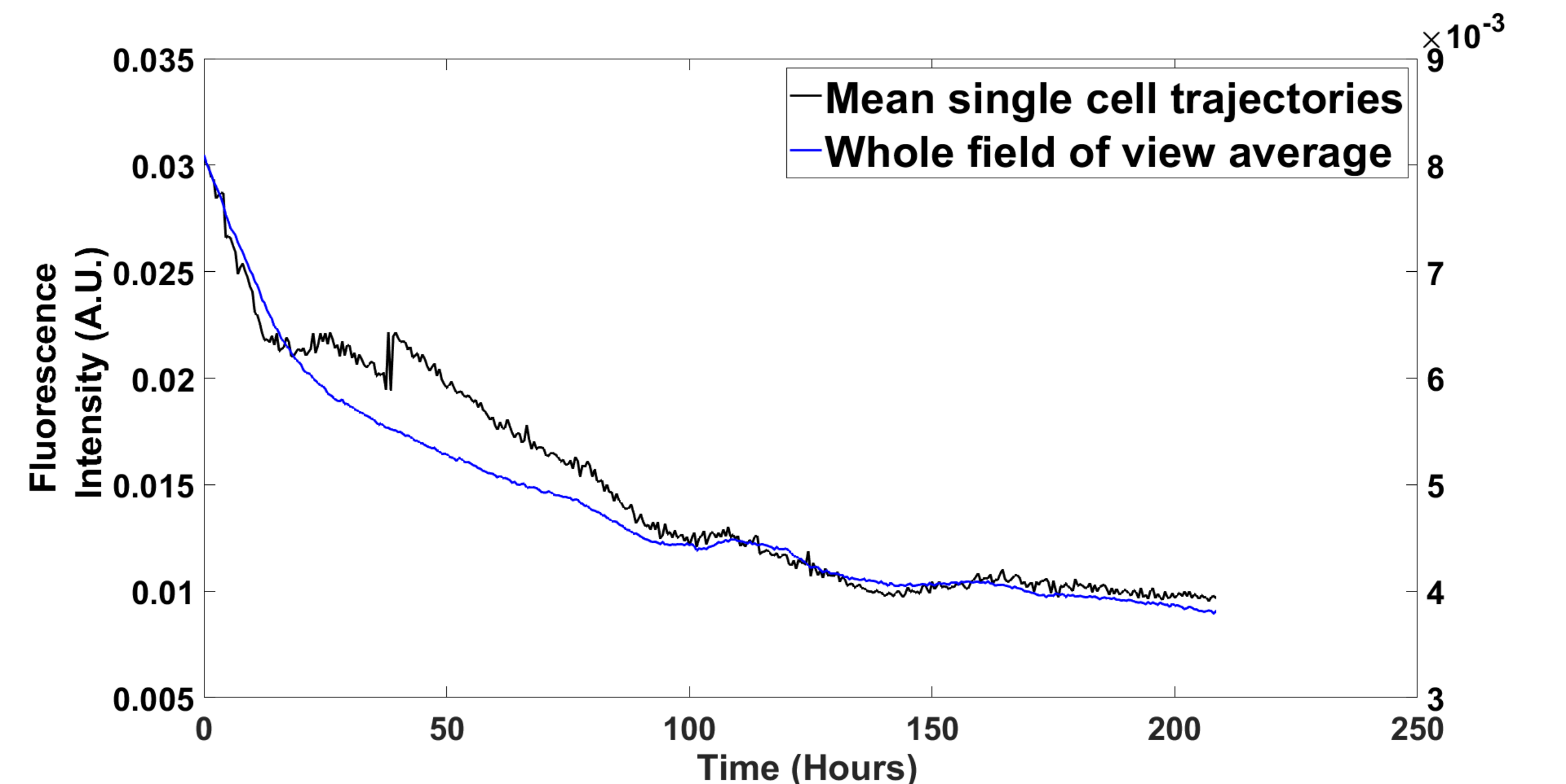


**Fig. S1** Mean single cell trajectories from the chamber microfluidic device tracked the whole field of view average intensity very well. **The plots were created in MATLAB_R2020B (**<https://www.mathworks.com/products/matlab.html>).


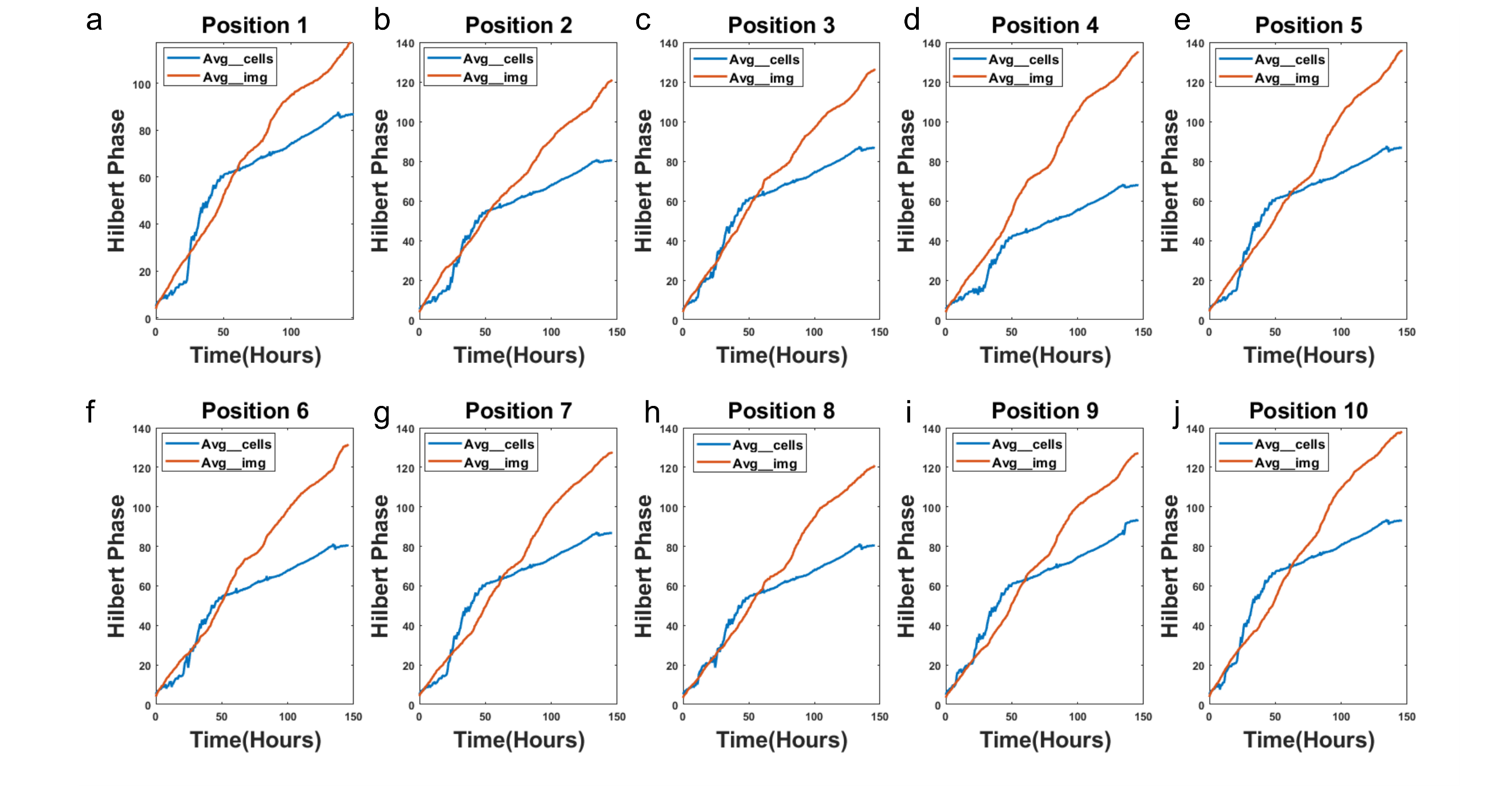


**Fig. S2** Average Phase of single cells tracks phase of a field of view across 10 fields of view in the big chamber device. The strain is MFNC9 (see Materials and Methods). Cells were grown in media 5 (see Materials and Methods) to block cell division. Fluorescence was measured every half hour over 10 days. **The plots were created in MATLAB_R2020B (**<https://www.mathworks.com/products/matlab.html>).


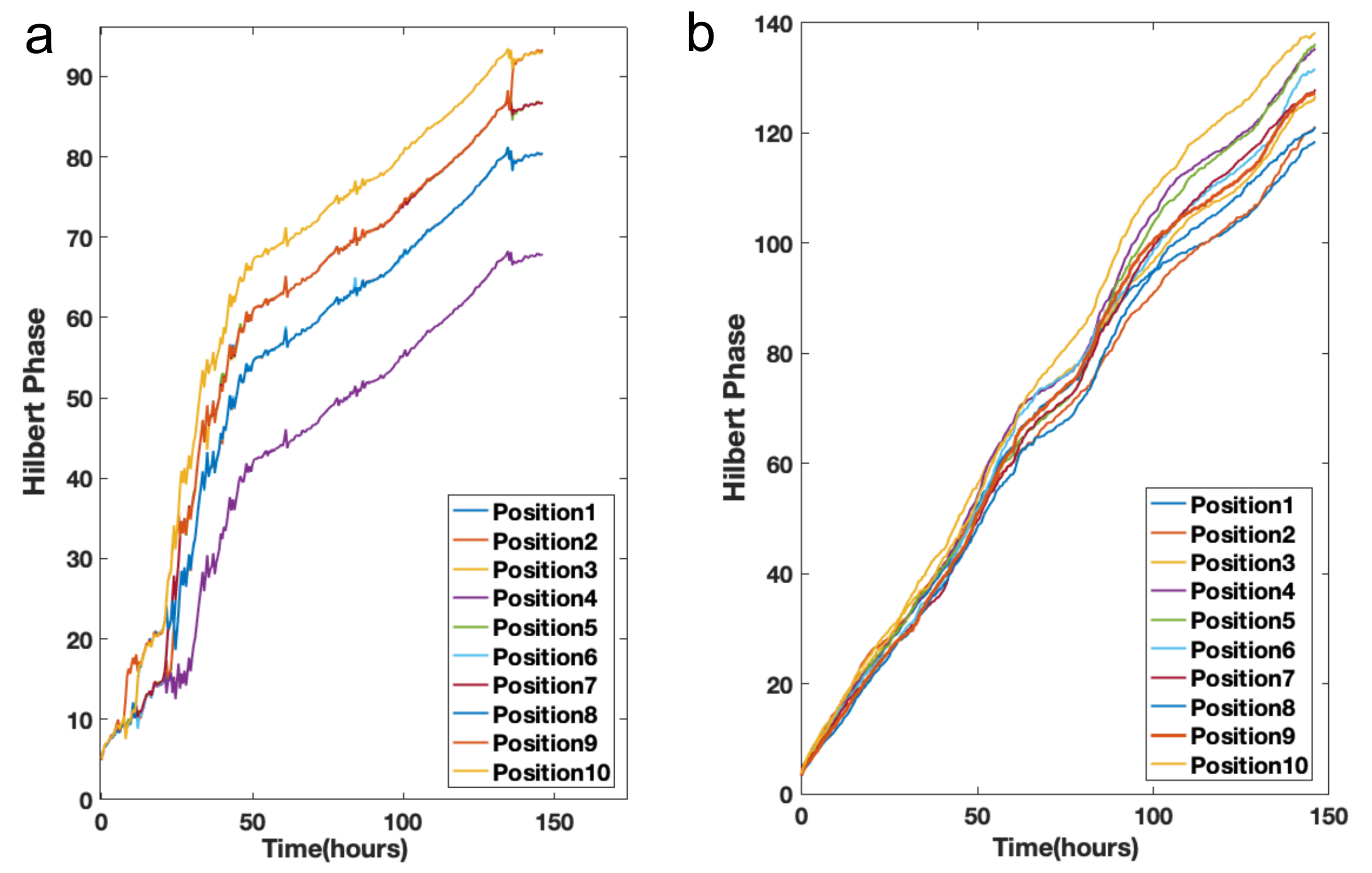


**Fig. S3** While the phase curves do track each other, they also fan out over time. The Hilbert phase curves from single cell trajectories were computed as described earlier^3^. (a) average of image (b) average of cells tracked. Single cell tracking was done with CellProfiler^4^. **The plots were created in MATLAB_R2020B (**<https://www.mathworks.com/products/matlab.html>).


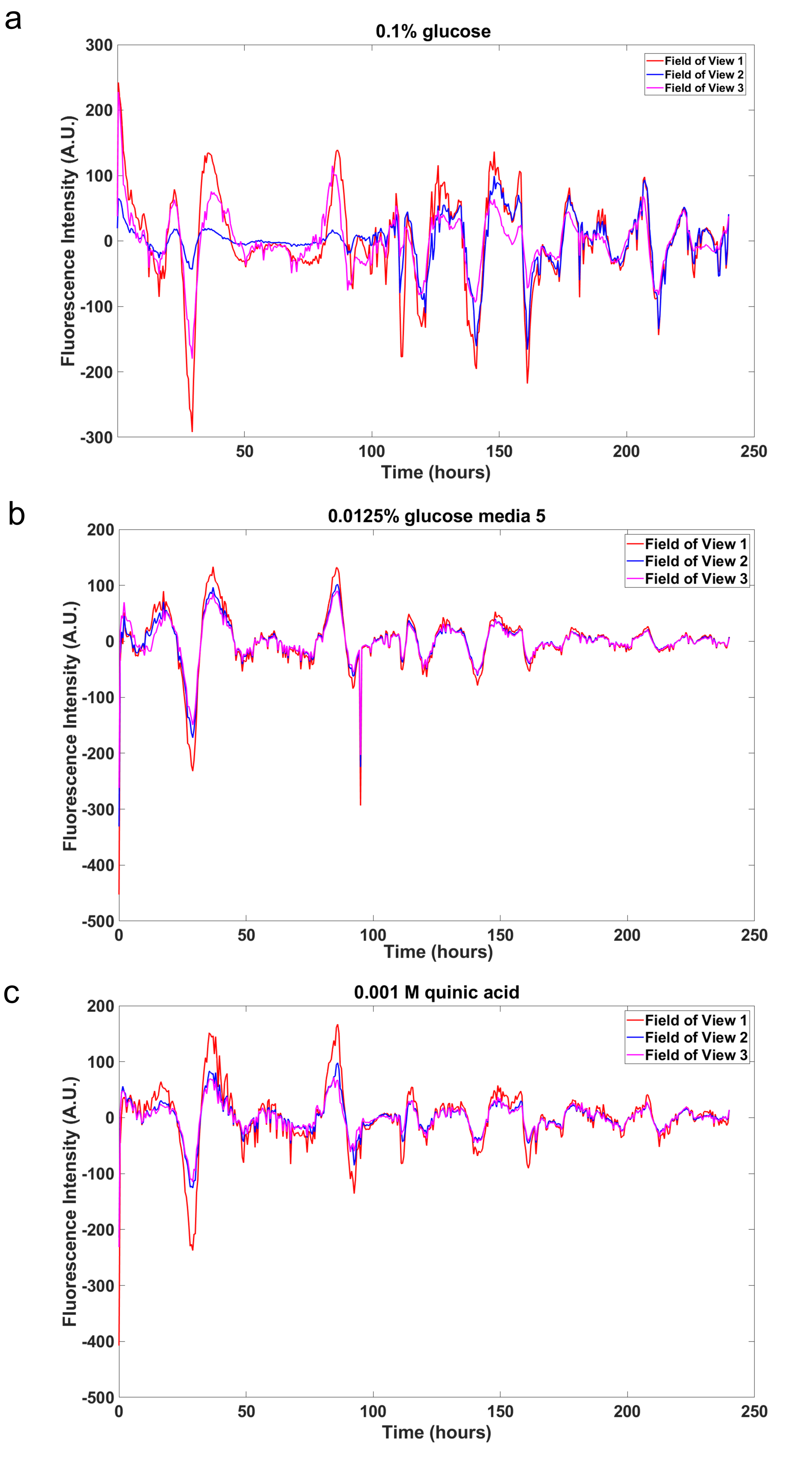


**Fig. S4** The fields of view in the big chamber device are highly synchronous in different media: (**a**) 0.1% glucose; (**b**) 0.0125% glucose; (**c**) 0.001 M quinic acid. **The plots were created in MATLAB_R2020B (**<https://www.mathworks.com/products/matlab.html>).


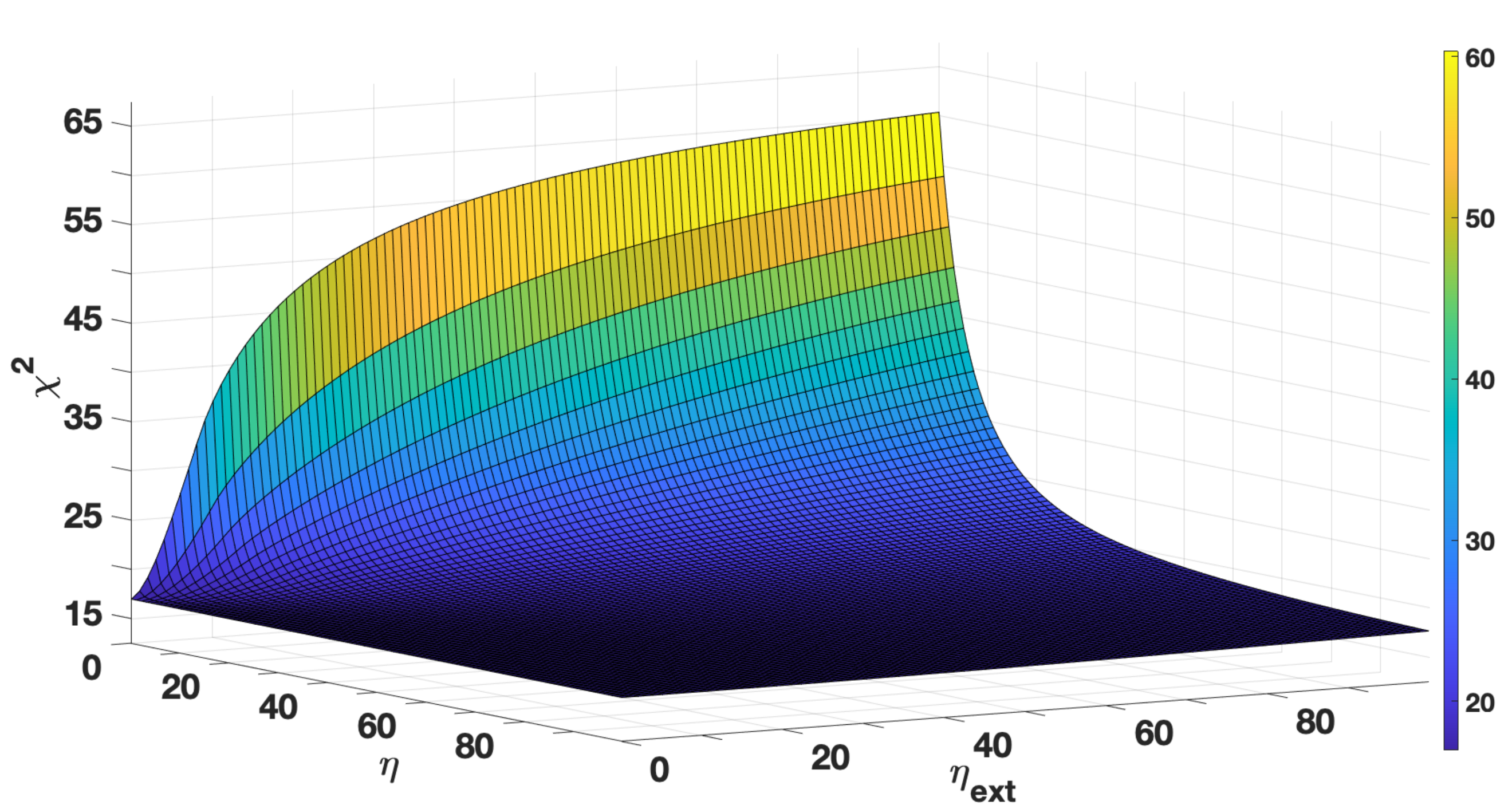


**Fig. S5** There is limited information about the diffusion parameters ($\eta$ and $\eta_{ext}$) for communication under the quorum sensing model as indicated by the chi-squared statistic surface as a function of $\eta$ and $\eta_{ext}.$For example, the surface is nearly flat with respect to $\eta_{ext}$. There is more information about $\eta$ – the surface increases sharply for $\eta_{ext}$around ~20 or below. Other parameters are set at their best values with respect to the chi-squared statistic in supplement Table S1. **The plots were created in MATLAB_R2020B (**<https://www.mathworks.com/products/matlab.html>).


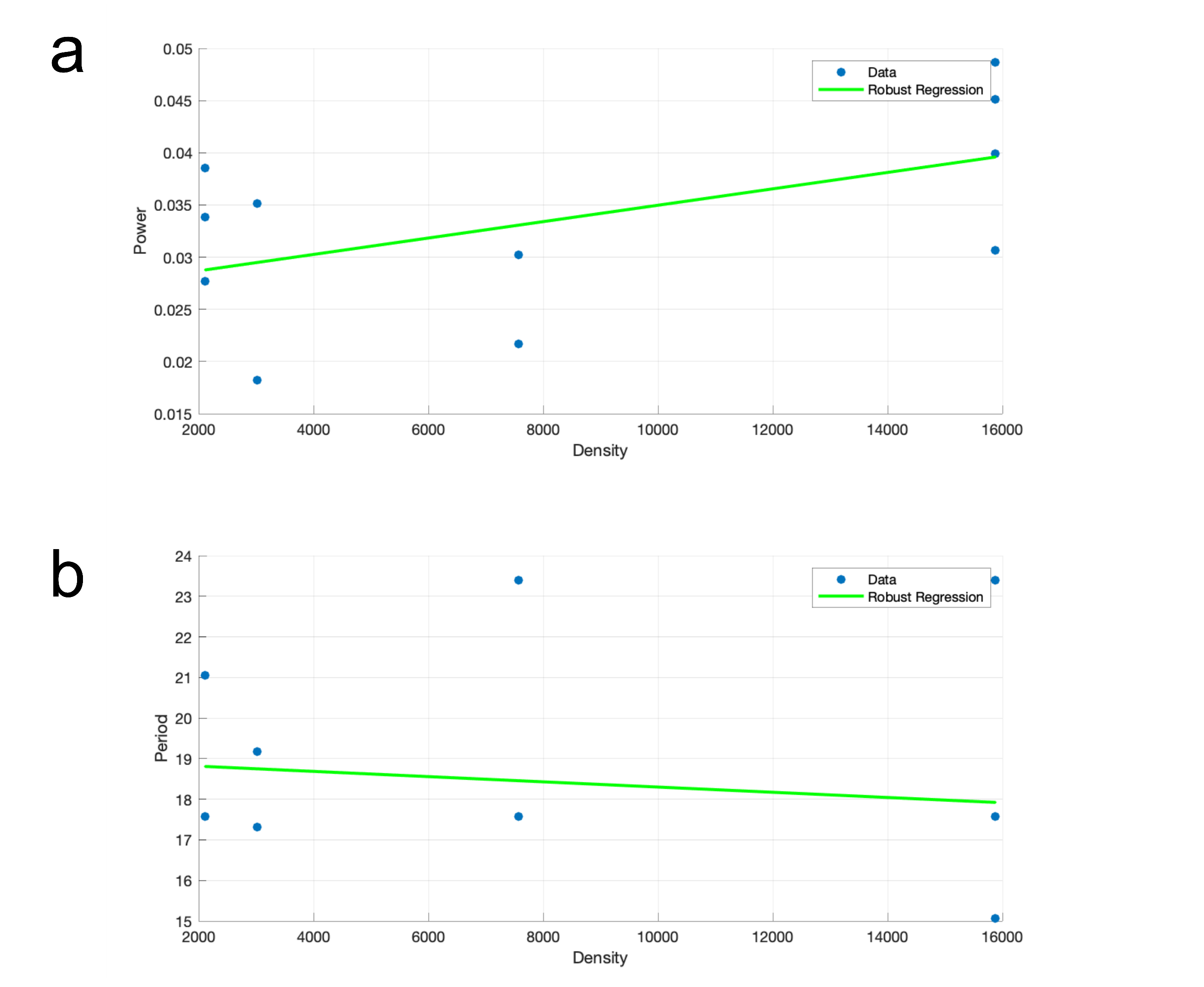


**Fig. S6 Relation between average Amplitude and Period of cell(s) in wells to Density in microwell device in Fig 11.** (**a**) Robust Regression of amplitude (as measured by the maximum in the periodogram) on number of cells for each microwell chamber using an M-estimator^64^ from 3 separate microwell experiments. The predicted regression line is amplitude = 0.03 + (7.86 $\pm$ 4.12)(10^-7^) x density (t_15_ = 1.9088, P = 0.04). At least 5,000 cells were tracked in each microwell experiment. (**b)** Robust Regression of Period on number of cells for each microwell chamber using an M-estimator^64^ from 3 separate microwell experiments. The slope of the predicted regression line is not significantly different from zero (t_15_ = -1.236, P = 0.55). At least 5,000 cells were tracked in each microwell experiment. All t-tests were one sided as in Fig. 11 for the sake of comparison to Fig. 11b.

**References**

1 Caranica, C., Al-Omari, A., Schuttler, H.-B. & Arnold, J. Identifying a clock stochastic network with light entrainment for single cells of *Neurospora crassa* by ensemble methods. *Nature Scientific Reports* **19**, 15168 (2020).

2 Dong, W. *et al.* Systems biology of the clock in Neurospora crassa. *PloS one* **3**, e3105 (2008).

3 Caranica, C. *et al.* What is phase in cellular clocks? *Yale Journal of Biology and Medicine* **92**, 169-178 (2019).

4 McQuin, C. *et al.* CellProfiler 3.0: Next-generation image processing for biology. *PLOS Biology* **16**, e2005970, doi:10.1371/journal.pbio.2005970 (2018).

5 Yu, Y. *et al.* A genetic network for the clock of *Neurospora crassa*. *Proc Natl Acad Sci USA* **104**, 2809-2814 (2007).
